# Identifying *Bixa orellana* L. New Carotenoid Cleavage Dioxygenases 1 and 4 Potentially Involved in Bixin Biosynthesis

**DOI:** 10.3389/fpls.2022.829089

**Published:** 2022-02-11

**Authors:** Rosa Us-Camas, Margarita Aguilar-Espinosa, Jacobo Rodríguez-Campos, Alba Adriana Vallejo-Cardona, Víctor Manuel Carballo-Uicab, Hugo Serrano-Posada, Renata Rivera-Madrid

**Affiliations:** ^1^Unidad de Bioquímica y Biología Molecular de Plantas, Centro de Investigación Científica de Yucatán A.C., Mérida, Mexico; ^2^Unidad de Servicios Analíticos y Metrológicos, Centro de Investigación y Asistencia en Tecnología y Diseño del Estado de Jalisco, Guadalajara, Mexico; ^3^Unidad de Biotecnología Médica y Farmacéutica, CONACYT, Centro de Investigación y Asistencia en Tecnología y Diseño del Estado de Jalisco, Guadalajara, Mexico; ^4^CONACYT, Laboratorio de Biología Sintética, Estructural y Molecular, Laboratorio de Agrobiotecnología, Colima, Mexico

**Keywords:** *BoCCD1*, *BoCCD4*, bixin aldehyde, norbixin, bixin biosynthesis, *Bixa orellana* L., apocarotenoids, carotenoids

## Abstract

Carotene cleavage dioxygenases (CCDs) are a large family of Fe^2+^ dependent enzymes responsible for the production of a wide variety of apocarotenoids, such as bixin. Among the natural apocarotenoids, bixin is second in economic importance. It has a red-orange color and is produced mainly in the seeds of *B. orellana*. The biosynthesis of bixin aldehyde from the oxidative cleavage of lycopene at 5,6/5′,6′ bonds by a CCD is considered the first step of bixin biosynthesis. Eight *BoCCD* (*BoCCD1-1, BoCCD1-3, BoCCD1-4, CCD4-1, BoCCD4-2, BoCCD4-3* and *BoCCD4-4*) genes potentially involved in the first step of *B. orellana* bixin biosynthesis have been identified. However, the cleavage activity upon lycopene to produce bixin aldehyde has only been demonstrated for BoCCD1-1 and BoCCD4-3. Using *in vivo* (*Escherichia coli*) and *in vitro* approaches, we determined that the other identified BoCCDs enzymes (BoCCD1-3, BoCCD1-4, BoCCD4-1, BoCCD4-2, and BoCCD4-4) also participate in the biosynthesis of bixin aldehyde from lycopene. The LC-ESI-QTOF-MS/MS analysis showed a peak corresponding to bixin aldehyde (*m/z* 349.1) in pACCRT-EIB *E. coli* cells that express the BoCCD1 and BoCCD4 proteins, which was confirmed by *in vitro* enzymatic assay. Interestingly, in the *in vivo* assay of BoCCD1-4, BoCCD4-1, BoCCD4-2, and BoCCD4-4, bixin aldehyde was oxidized to norbixin (*m/z* 380.2), the second product of the bixin biosynthesis pathway. *In silico* analysis also showed that BoCCD1 and BoCCD4 proteins encode functional dioxygenases that can use lycopene as substrate. The production of bixin aldehyde and norbixin was corroborated based on their ion fragmentation pattern, as well as by Fourier transform infrared (FTIR) spectroscopy. This work made it possible to clarify at the same time the first and second steps of the bixin biosynthesis pathway that had not been evaluated for a long time.

## Introduction

Carotenoids comprise a broad group of mostly colored molecules on which the oxidative cleavage of carotenoid cleavage oxygenases [CCOs — also know as carotenoid cleavage dioxygenases (CCDs)] — act to produce numerous derivatives known as apocarotenoids. Some of these apocarotenoids have color, such as bixin, a red-orange monomethyl ester of the C_24_ dicarboxylic acid norbixin, that is accumulated mainly in the seeds of *Bixa orellana* (achiote or annatto-tree) (Rivera-Madrid et al., 2016). Achiote is a perennial tree species native to the Amazonia and tropical regions (Akshatha et al., 2011; Moreira et al., 2015). Its economic importance lies in the high content of bixin in its seeds (about 80% of total carotenoids). Bixin is a red-orange apocarotenoid widely used in the food, pharmaceutical, cosmetic, and textile industries (Tirimanna, 1981; Giuliano et al., 2003; Rivera-Madrid et al., 2006, 2016). The demand for bixin has increased in recent years due to its potential use as a natural dye and its health and environmental benefits over synthetic dyes (Evans, 2000; Devi et al., 2013; Shahid ul et al., 2016). The apocarotenoid bixin is produced by the oxidative cleavage at the double bonds of the carotenoid polyene chain by the action of the carotenoid cleavage dioxygenases (CCDs), a large family of non-iron (II) dependent enzymes present in all taxa (Auldridge et al., 2006; Hou et al., 2016; Ramamoorthy et al., 2020). In plants, CCDs are divided into two functionally different groups: the 9-*cis* epoxycarotenoid dioxygenases (NCEDs) and the CCDs (Walter et al., 2010). NCEDs participate in the biosynthesis of ABA through cleavage at the 11,12 double bonds of 9-*cis*-violaxanthin and 9-*cis*-neoxanthin to produce xanthoxin (C_15_), the ABA precursor (Tan et al., 2003; Us-Camas et al., 2020). CCDs have recently been classified into six subfamilies: CCD1, CCD2, CCD4, CCD7, CCD8, and ZAS (zaxinone synthase), which vary in their substrate specificity and cleavage sites (Auldridge et al., 2006; Frusciante et al., 2014; Hou et al., 2016; Fiorilli et al., 2019; Rivera-Madrid and Ramamoorthy, 2020). *In vitro* analyzes indicate that CCD1 enzymes can cleave different cyclic and linear carotenoids at the 5,6/5′,6′, 7,6/7′,6′, and 9,10/9′,10′ double bonds, generating a great diversity of apocarotenoids as products (Schwartz et al., 2001; Mathieu et al., 2005; Rubio-Moraga et al., 2008; Vogel et al., 2008; Ilg et al., 2009; Lashbrooke et al., 2013). In planta, CCD1 cleaves C_27_ apocarotenoid at 9,10/9′,10′ positions to produce volatile C_13_ (β-ionone) and C_14_ (dialdehydes) apocarotenoids involved in flavor, aroma, and root colonization (Floss et al., 2008; Floss and Walter, 2009). On the other hand, CCD4 enzymes are capable of cleaving the 5,6/5′,6′, 7,6/7′,6′, and 9,10/9′,10′ positions of different carotenoids (Bouvier et al., 2003; Rubio-Moraga et al., 2008; Lashbrooke et al., 2013; Ma et al., 2013; Rodrigo et al., 2013). CCD4 enzymes are mainly involved in the regulation of volatile apocarotenoids biosynthesis (Rubio-Moraga et al., 2008; Brandi et al., 2011; Lashbrooke et al., 2013), as well as in the regulation of pigmentation of flower petals, fruits, and seeds (Ohmiya et al., 2006; Campbell et al., 2010; Brandi et al., 2011; Gonzalez-Jorge et al., 2013; Rodrigo et al., 2013; Ko et al., 2018). CCD4 enzymes generally have a chloroplast transit peptide in their sequence. In contrast, CCD1 enzymes are located in the cytosol (Rubio-Moraga et al., 2008). Some CCD4 enzymes are associated with the plastoglobules of chloroplasts, where they perform carotenoid cleavage (Ytterberg et al., 2006; Rottet et al., 2016).

**FIGURE 1:**
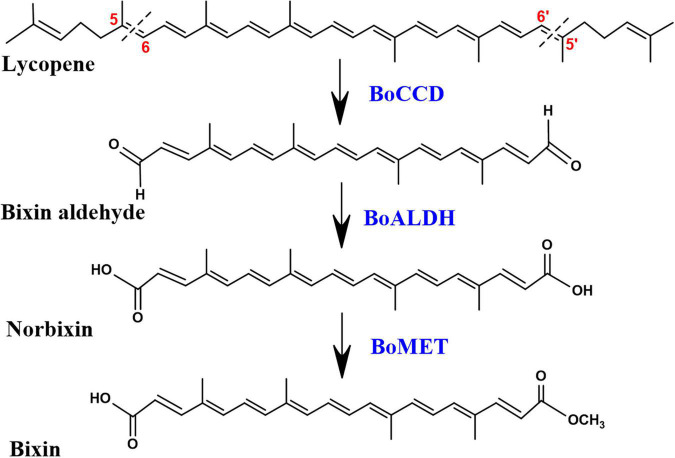
Bixin biosynthesis pathway. All compounds are in the *trans* configuration. BoCCD, carotene cleavage dioxygenase; BoALDH, bixin aldehyde dehydrogenase; BoMET, norbixin methyltransferase.

The investigation carried out by Bouvier et al. (2003) demonstrated that bixin biosynthesis begins with the oxidative cleavage of C_40_ lycopene at the 5,6/5′,6′ double bonds by the CCD4 enzyme lycopene dioxygenase (BoLCD), to produce bixin aldehyde. Subsequently, the aldehyde groups are oxidized to carboxyls by bixin aldehyde dehydrogenase (BoALDH) producing norbixin, and finally, bixin is produced when one of the carboxyl group of norbixin is methylated by norbixin methyltransferase (BoMTH) (Jako et al., 2002; Bouvier et al., 2003; Figure 1). The sequential activity of these three enzymes in lycopene-producing *Escherichia coli* cells produced bixin (Bouvier et al., 2003). However, the *BoLCD* (CCD4 type) sequence, as well as the *BoALDH* and *BoMTH* genes involved in bixin biosynthesis (Bouvier et al., 2003) were not found in the leaves nor in mature and immature seed transcriptomes of the local Yucatecan accession “Peruana Roja” of *B. orellana* (Cárdenas-Conejo et al., 2015). Instead, other members of the *BoCCDs*, *BoALDHs* and, *SABATHs* (methyltransferases) gene families potentially involved in the bixin biosynthesis were identified. Derived from achiote transcriptomes analysis, Cárdenas-Conejo et al. (2015) identified eight candidate *BoCCDs* genes involved in bixin biosynthesis, distributed in hypothetical cytosolic and plastidic pathways, four of them belonging to the *BoCCD1* subfamily: *BoCCD1-1*, *BoCCD1-2*, *BoCCD1-3*, and *BoCCD1-4*; and four of them to the *BoCCD4* subfamily: *BoCCD4-1*, *BoCCD4-2*, *BoCCD4-3* and, *BoCCD4-4* (Cárdenas-Conejo et al., 2015). Recently, Carballo-Uicab et al. (2019) demonstrated that the recombinant proteins BoCCD1-1 and BoCCD4-3 exhibited cleavage activity at the 5,6/5′,6′ positions of lycopene producing bixin aldehyde, indicating their involvement in the first step of bixin biosynthesis (Carballo-Uicab et al., 2019). Other *BoCCD1* and *BoCCD4* genes have been isolated from different *B. orellana* cultivars, but their role in bixin biosynthesis has not been unveiled (Rodríguez-Ávila et al., 2011b; Soares et al., 2011; Sankari et al., 2016). In general, the expression levels of all *BoCCD* genes identified in the transcriptomes increased in the immature stages of the seed (S3 and S4) when bixin reached its maximum levels and decreased in the mature stages of the seed (S5) when bixin levels dropped dramatically in the two accessions that produce contrasting levels of bixin (N4P and P13W) (Carballo-Uicab et al., 2019). Therefore, we hypothesize spatio-temporal participation in bixin biosynthesis of all the BoCCD1 and BoCCD4 enzymes identified in the transcriptomes of *B. orellana*. Furthermore, it has been suggested that the BoCCD1 and BoCCD4 enzymes are distributed in two hypothetical pathways, a cytosolic and a plastidic, respectively, where they can act individually or in coordination to synthesize bixin, reinforcing our hypothesis (Cárdenas-Conejo et al., 2015). For this reason, in the present study, we used different approaches to determine if other BoCCD1 and BoCCD4 enzymes, in addition to BoCCD1-1, and BoCCD4-3, have 5/6, 5′/6′ cleavage activity on lycopene and if they are involved in the first step of bixin biosynthesis.

We isolated, cloned, and expressed seven of the eight *BoCCD* genes reported in *B. orellana*; three of them belong to the *BoCCD1* subfamily: *BoCCD1-1*, *BoCCD1-3*, and *BoCCD1-4*; and four belonging to the *BoCCD4* subfamily: *BoCCD4-1*, *BoCCD4-2*, *BoCCD4-3*, and *BoCCD4-4*. Sequence alignment and phylogenetic analysis indicated that all isolated BoCCD1 and BoCCD4 proteins encode functional carotenoid cleavage dioxygenases. The production of bixin aldehyde (*m/z* 349) from lycopene was confirmed by Liquid Chromatography Electrospray Ionization Quadrupole Time-of-Flight Mass Spectrometry (LC-ESI-QTOF-MS/MS) analyzes in the *in vivo* (*E. coli*) assay. *In vitro* enzymatic assays indicated that in addition to BoCCD1-1 and BoCCD4-3; BoCCD1-3, BoCCD1-4, BoCCD4-1, BoCCD4-2, and BoCCD4-4 enzymes have cleavage activity at the 5,6/5′,6′ double bonds of lycopene. Three-D structural modeling and docking analyzes also indicated that all the isolated BoCCD1 and BoCCD4 proteins can use lycopene as a substrate. Interestingly, we found that in the *in vivo* assay of BoCCD1-4, BoCCD4-1, BoCCD4-2, and BoCCD4-4, bixin aldehyde (*m/z* 349.1) was oxidized to norbixin (*m/z* 380.2), the second product of the bixin biosynthesis pathway. Our results suggest that the second product of bixin biosynthesis pathway, norbixin, can be generated by an oxidation process in *E. coli* cells, but further studies are required. The production of bixin aldehyde and norbixin were corroborated based on their ion fragmentation patterns and by Fourier transform infrared (FTIR) spectroscopy analysis. This work allowed the identification of new BoCCD enzymes potentially involved in the first step of bixin biosynthesis in *B. orellana* and at the same time clarified the first and second step of bixin biosynthesis pathway that had not been evaluated for a long time.

## Materials and Methods

### Plant Material

The study used *B. orellana* N4P accession, which has distinctive morphological characteristics: pink flowers, dehiscent green fruits with red spines, and seeds with high bixin contents (Trujillo-Hdz et al., 2016). Young leaves from the N4P accession were collected and immediately immersed in liquid nitrogen then stored at −80°C until subsequent RNA extraction.

### Total RNA Extraction and cDNA Synthesis

Total RNA was extracted from the leaves of *B. orellana* using the PureLink RNA Mini Kit (Cat. No. 12183018A, Invitrogen) as described by Rodríguez-Ávila et al. (2009). First-strand cDNA was synthesized from 1 μg of total RNA using the SuperScriptTM III reverse transcriptase (Cat. No. 18080-093) according to the manufacturer’s instructions.

### Isolation and Cloning of *BoCCD1* and *BoCCD4* Genes

The coding region of *BoCCD1-1*, *BoCCD1-3*, *BoCCD1-4*, *BoCCD4-1*, *BoCCD4-2*, *BoCCD4-3*, and *BoCCD4-4* was isolated from leaf cDNA of the N4P accession of *B. orellana* using the primers listed in Supplementary Table 1. The primers were designed using the sequences of the *BoCCDs* found in the transcriptome and reported in the GeneBank (Cárdenas-Conejo et al., 2015). The PCR reactions were performed using the Phusion High-Fidelity DNA Polymerase (Cat. No. F534S, Thermo Fisher Scientific). The reaction contained 0.5 μl of cDNA at ∼1000 ng/μl, 4 μl of 5X Phusion Green HF Buffer, 0.1 μl of MgCl_2_ at 50 mM, 0.4 μl of a mix of dNTPs at 10 mM, 0.5 μl of specific forward and reverse primers at 10 Mm, 0.1 μl of Phusion High-Fidelity DNA Polymerase at 2 U/μl, and sterile water to a final volume of 20 μl. PCR reactions were carried out with the following program: initial denaturation at 98°C for 30 s, denaturation at 98°C for 15 s, alignment for 30 s at the temperature indicated in Supplementary Table 1, extensión at 72°C for 30 s for 35 cycles, and a final extension at 72°C for 10 min. The PCR products were resolved on SYBR Green I-stained agarose gels (Cat. No. S7563, Thermo Fisher Scientific), and the fragments with the expected sizes (Supplementary Figure 1) were purified using the QIAquick gel extraction kit (Cat. No. 27104, QIAGEN), following the supplier’s specifications. The individually purified ORFs were subjected to a 3′A addition reaction and subsequently cloned into the vector pCR8/GW/TOPO (Cat. No. K250020, Thermo Fisher Scientific). DH5α cells were transformed, and the positive colonies were selected in semisolid Luria–Bertani (LB) media supplemented with spectinomycin (100 μg/ml). Finally, the ORFs were recombined in-frame to the N-terminal HIS-tag of the pDEST17 expression vector (Cat. No. 11803-012, Thermo Fisher Scientific), using the Gateway Technology (Cat. No. 11791020, Thermo Fisher Scientific). The transformed DH5α cells were selected using ampicillin (100 μg/ml). The correct cloning of the ORFs was confirmed by enzymatic digestion and sequencing.

### Bioinformatic Analysis

The pDEST17-*BoCCDs* plasmids were sequenced (Macrogene, Seoul, South Korea), and the identity of the sequences was confirmed by comparing them with the NCBI sequence database, using the Basic Local Alignment Search Tools (BLASTn and BLASTp)^1^. The identity and similarity scores of the sequenced coding regions of all BoCCDs with their homologs reported in the GeneBank from the transcriptome were estimated using the Clustal W program with the default settings^2^. Amino acid sequences were obtained using the translate tool of Expasy server^3^. Multiple alignments of BoCCD1 and BoCCD4 sequences were performed using the Clustal W algorithm and the DNAMAN program. The potential subcellular location of the BoCCDs were predicted using the Ipsort^4^, ProtComp 9.0^5^, and PREDATOR^6^ programs.

### Phylogenetic Analysis

The phylogenetic tree was constructed using the amino acid sequences of all BoCCD1 and BoCCD4 proteins, and other functionally characterized CCD1 and CCD4 protein sequences from different plants species. Most of the sequences were retrieved from the GeneBank and some from Sol Genomics Network databases^7^. The phylogenetic tree was constructed using the Maximum likelihood method based on Jones-Taylor-Thornton (JTT) substitution model and Gamma distributed with Invariant sites (G + I), using 1000 bootstrap through MEGA X software (Kumar et al., 2018). The gaps and missing data were partially eliminated. The amino acid sequences were aligned using CLUSTAL W with the default parameter on MEGAX. The substitution model was estimated using the best-fit substitution model (ML) function included in MEGA X.

### 3D Modeling and Docking Analysis

The 3D structure of the BoCCD1 and BoCCD4 proteins were modeled with the online software PHYRE2^8^ using a profile-profile alignment algorithm against the fold library of PHYRE2. Sequence alignment was performed using the online version of CLUSTALW. For docking analysis, Fe^+2^ coordinates in each CCD model complex were selected to define the center of a 25 Å cubic grid box for the search for the geometry of the lycopene docking. Lycopene ligand (zinc8214943) was selected from the Zinc database^9^. The initial geometry was optimized by minimizing energy with the Yasara software^10^ and eliminating hydrogens with the Phenix program. As the final model of each complex, the one with the lowest protein-ligand affinity free energy was selected. Structures and docking calculations were obtained using AutoDock Vina. Finally, the analysis of the structures and the visualization were carried out with UCSF Chimera 1.11, and Discovery studio.

### SDS-PAGE and Protein Blot Analysis

Five milliliters of the cultures grown at 37°C for 20 h at 175 rpm and induced with IPTG at a final concentration of 0.5 mM were pelleted and solubilized with 8 M urea for 10 min at 96°C. The amount of total protein was equalized with urea 8 M based on culture optical density OD_600_. Sixteen μl of the solubilized sample were mixed with 4 μl of 5X loading buffer and boiled for 6 min at 96°C. Samples were loaded on 10% SDS/PAGE gels and run in a Mini-PROTEAN Tetra Cell chamber. Protein bands were stained with comasie blue or transferred to a nitrocellulose membrane. Protein blot analysis was performed following the procedure described by Nic-Can et al. (2013), using the anti-His tag (6E6) monoclonal antibody and the HRP conjugate as a secondary antibody. The signal was detected using the commercial ECL chemiluminescence solution.

### *In vivo* Expression of BoCCD1 and CCD4 Proteins

BL21 (DE3) competent *E. coli* cells were cotransformed with the plasmid pACCRT-EIB that produces lycopene and pDEST17-*BoCCDs* plasmids. The negative controls were BL21 (DE3) cells containing the empty pDEST17 vector. Double recombinant cells were selected in semi-solid LB medium with ampicillin (100 μg/ml) and chloramphenicol (34 μg/ml). Two positive colonies were placed in 6 ml of liquid LB medium with the same antibiotics at the same concentrations and were incubated overnight at 37°C at 200 rpm. Two milliliters of the overnight cultures were transferred into 100 ml of LB liquid medium with the same antibiotics at the same concentrations until they reached an OD_600_ of 0.4-0.5. Protein expression was induced with IPTG at a final concentration of 0.5 mM. Cultures were incubated in dark conditions at 37°C for 20 h at 175 rpm. Cells were pelleted in two tubes of 50 ml at 6000 g (Universal 32 R; Hettich zentrifugen, Tuttlingen, Germany) for 20 min at 4°C. For pigment extraction, the pellets were resuspended in 1.7 ml of cold 2:1 chloroform/methanol, centrifuged at 14000 rpm for 20 minutes at 4°C, and the organic phase was placed in a new tube. The organic phase was filtered (Durapore PVDP, 13 mm diameter, 0.22 μm of size, Millipore) and subsequently dried under a stream of nitrogen. All steps were performed on ice and under dark conditions. Each sample was stored at −80°C until it was analyzed by mass spectrometry to identify the compounds.

### *In vitro* Functional Assay of BoCCD1 and BoCCD4 Proteins

For *in vitro* analysis the *PDEST17-BoCCDs* constructs were transformed into BL21 (DE3) cells. One milliliter of the overnight cultures was transferred into 50 ml of LB liquid medium with half-strength of the antibiotic ampicillin (50 μg/ml), incubated at 37°C at 200 rpm until reaching an OD_600_ of 0.4-0.5. Protein expression was induced with IPTG at a final concentration of 0.5 mM, at 25°C for 20 h at 175 rpm. The crude extracts and the *in vitro* reactions were prepared as described by Vogel et al. (2008) with slight modifications. Fifty milliliters of the growth pellet were resuspended in 4.5 ml of cold phosphate-buffered saline (PBS) with 1 mM of phenylmethylsulfonyl fluoride. Samples were sonicated four times for 30 s at máximum power on ice, and then 0.5 ml of 20% Triton X-100 in PBS buffer was added. The cell slurry was shaken for 20 min on ice and then centrifuged at 12,000 × g for 20 min at 4°C. The soluble fractions were filtered through a 0.22 μm filter, and then glycerol was added to a final concentration of 20%; the lysate was aliquoted and stored at −20°C. To obtain an aqueous solution for the enzymatic reactions, lycopene standard was prepared by adding 1% of Tween 40 in chloroform. The solution was vigorously vortexed, and 60 ppm aliquots were removed for each reaction. The aliquots were dried under a stream of nitrogen. The *in vitro* reaction was carried out in a final volume of 1 ml containing 50 mM NaH_2_PO_4_ (pH 7.2), 300 mM NaCl, 5 μM FeSO_4_, 5 Mm ascorbic acid, and 200 μl of the total protein extract. The reactions were incubated for 20 h at 30°C and stopped by adding chloroform/methanol (3:1). Compounds were extracted as previously described.

### LC-ESI-QTOF-MS/MS

The mass spectra of the different compounds were obtained on a QTOF Xevo G2-S (Waters Corporation, Manchester, UK) with an electrospray source in positive ion mode. Bacterial pellets were resuspended in 100 μl of dichloromethane. Fifty microliters were diluted in 950 μl of isopropanol: acetonitrile (1:1.375). The samples were injected into the mass spectrometer by direct infusion, and the sample infusion flow rate was 15 μL/min. The conditions of the ionization were optimized as follows: the source temperature and desolvation temperatures were 100°C and 400°C, respectively. The desolvation and cone gas flow were 800 and 50 L/h, respectively. The cone and capillary voltages were 30 and 4,000 V, respectively. For the MS/MS acquisition, the collision energies were 6 and 15 eV. The acquisition mass range was 50 to 1500 *m/z* with a scan time of 1 s and resolution mode.

### FTIR Analysis

The functional groups of the oxidative cleavage products of the *in vitro* and *in vivo* assay of BoCCD1 and BoCCD4 protein expression were determined by Fourier transform infrared (FTIR) absorption spectra (range 3600 to 500 cm^–1^) using a TENSOR II FTIR Spectrometer (Bruker Corporation) with default settings. For the analyzes, 20 μl of the dissolved bacterial extracts were mixed with 20 μl of Milli-Q water. Standard lycopene was used as a control. Two microliters were used for each analysis.

## Results

### Sequence Analysis of BoCCD1 and BoCCD4 Proteins Reveals They Preserve the Active Site Structure of the Carotene Cleavage Dioxygenases

The full-length cDNA of *BoCCD1-1*, *BoCCD1-3*, *BoCCD1-4*, *BoCCD4-1*, *BoCCD4-2*, *BoCCD4-3*, and *BoCCD4-4* genes were amplified from *B. orellana* mRNA using specific primers (Supplementary Table 1). Fragments with the expected sizes were cloned and sequenced (Supplementary Figure 1). The cloned genes were sequenced, and the identities of all isolated *BoCCD*s genes were confirmed by comparing them with their homologous sequences obtained from the transcriptome and reported in the Genebank (Cárdenas-Conejo et al., 2015). Sequence analysis of the members of the *BoCCD1* family showed that *BoCCD1-1* contains an open frame of 1,629 nucleotides (nt) and encodes a predicted protein of 542 amino acids (aa); *BoCCD1-3* contains 1,644 nt and encodes a protein of 547 aa, and *BoCCD1-4* contains 1,515 nt and encodes a protein of 504 aa. The analysis of the members of the *BoCCD4* family showed that *BoCCD4-1* contains 1,800 nt and encodes a predicted protein of 599 aa, *BoCCD4-2* contains 1,749 nt and encodes a protein of 582 aa, *BoCCD4-3* contains 1, 773 nt and encodes a protein of 590 aa and finally, *BoCCD4-4* contains 1, 863 nt and encodes a protein of 620 aa (Supplementary Table 2). The open reading frame of all the isolated *BoCCDs* showed changes at the nucleotide level in their sequence that generated changes at the amino acid level without altering the size of their open reading frame. Only BoCCD4-3 presented the insertion of a proline in position 72 (Supplementary Figure 2). Additionally, we found that the missing nucleotide in the *BoCCD4-3* sequence reported in the Genebank (accession number: KT359024.1) corresponds to an adenine, and the complete codon encodes a lysine (Supplementary Figure 2). Finally, the level of homology between the isolated genes and those reported in the transcriptome were > 98%, and 97% at the nucleotide and amino acid levels, respectively (Supplementary Table 2), indicating that the isolated *BoCCD1* and *BoCCD4* genes are those previously reported.

To elucidate the phylogenetic relationship of the isolated BoCCD1 and BoCCD4 proteins, a phylogenetic analysis of the deduced BoCCD protein sequences with other functionally characterized CCD1 and CCD4 proteins from several plant species was performed. The protein sequence of *Synechocystis* sp. apocarotenoid cleavage oxygenase (ACO) was used as an external group (Supplementary Figure 3). The results show the formation of two major clades corresponding to the CCD1 and CCD4 subfamilies of enzymes. As expected, the results confirmed that the isolated BoCCD1-1, BoCCD1-3, and BoCCD1-4 belong to the CCD1 subfamily; and BoCD4-1, BoCCD4-2, BoCCD4-3, and BoCCD4-4 belong to the CCD4 subfamily (Supplementary Figure 3). Supplementary Figure 3 also shows that the BoLCD (Accession No. CAD33263.1) published by Bouvier et al. (2003) is grouped with the CCD4 enzymes of *Crocus sativus* and is located outside the subgroup formed by the BoCCD4 enzymes, as previously reported (Cárdenas-Conejo et al., 2015). Interestingly, the BoCCD4 enzymes are closely related to citrus CCD4 enzymes, determinants of the reddish-orange color of the flavedo of citrus and mandarins fruits through the biosynthesis of the C_30_ apocarotenoid β-citraulin by cleaving zeaxanthin and β-cryptoxanthin at the 7/8 or 7′/8′ positions (Ma et al., 2013; Rodrigo et al., 2013; Zheng et al., 2019). Citrus CCD4b was also able to cleave lycopene at 7/8 and 5/6 positions producing the C_30_ apocarotenoid apo-8′-lycopenal and the C_27_ apocarotenoid apo-10′-lycopenal, respectively. CCD4b can also cleave apo-8′-lycopenal and apo-10′-lycopenal at 5/6 positions producing C_22_ and C_19_ dialdehydes, respectively (Rodrigo et al., 2013). BoCCD1 proteins formed two close subgroups; in the first subgroup, BoCCD1-1 showed to be more closely related to the BoCCD1 described by Rodríguez-Ávila et al. (2011b) and obtained from the same accession of *B. orellana*; in the second subgroup BoCCD1-3 and BoCCD1-4 were grouped together (Supplementary Figure 3; Cárdenas-Conejo et al., 2015; Carballo-Uicab et al., 2019).

To determine if the seven isolated BoCCDs conserved the active site residues necessary for dioxygenase activity, we performed an alignment using the predicted amino acid sequence of the isolated BoCCDs and the sequences of the CCD1 and CCD4 enzymes of *Arabidopsis thaliana* and *C. sativus* functionally characterized (Supplementary Figure 4). The alignment analysis revealed that the seven BoCCD contain four highly conserved histidine residues, as a Fe^+^-ligating cofactor, characteristic of the active site, two conserved glutamic acid residues, and one semi-conserved aspartate residue involved in fixing the iron-linked histidine residues (Harrison and Bugg, 2014; Daruwalla and Kiser, 2020; Supplementary Figure 4). According to the consensus of protein subcellular localization prediction programs, all BoCCD4 proteins contain the characteristic chloroplast transit peptide in the N-terminal region. In contrast, all BoCCD1 proteins appear to be cytosolic (Cárdenas-Conejo et al., 2015; Carballo-Uicab et al., 2019; Supplementary Table 3). Overall, these results suggest that the isolated *BoCCD1-1*, *BoCCD1-3*, *BoCCD1-4*, *BoCCD4-1*, *BoCCD4-2*, *BoCCD4-3*, and *BoCCD4-4* genes encode functional enzymes that have conserved their role as carotene cleavage dioxygenases in carotenoid metabolism.

### BoCCD1 and BoCCD4 Enzymes Are Involved in Bixin Aldehyde Biosynthesis

To determine the participation of the BoCCDs in the first step of bixin biosynthesis, the *in vivo* lycopene cleavage activity of BoCCD1-1, BoCCD1-3, BoCCD1-4, BoCCD4-1, BoCCD4-2, BoCCD4-3, and BoCCD4-4 was analyzed. The coding regions of the seven *BoCCDs* were recombined, producing a fused N-terminal HIS-tag protein in the pDEST17 expression vector, providing an IPTG-inducible expression in *E. coli*. The empty pDEST17 vector was used as a negative control. SDS/PAGE analysis showed that BoCCD1-1, BoCCD1-3, BoCCD1-4, BoCCD4-1, BoCCD4-2, BoCCD4-3, and BoCCD4-4 fusion proteins were expressed with the addition of IPTG, showing an apparent molecular mass of 60.97 kDa, 61.98 kDa, 57.42 kDa, 67.99 kDa, 65.88 kDa, 66.13 kDa, and 69.74 kDa, respectively (Supplementary Figures 5A,B). Protein blot analysis using anti-His antibody confirmed the expression of the seven BoCCDs (Supplementary Figure 5C).

**FIGURE 2:**
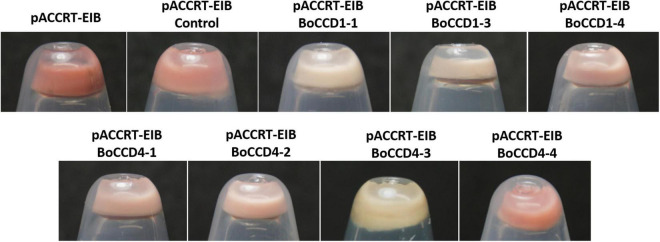
Discoloration of pCCART-EIB *E. coli* cells that accumulate lycopene by the expression of BoCCD1-1, BoCCD1-3, BoCCD1-4, BoCCD4-1, BoCCD4-2, BoCCD4-3, and BoCCD4-4 proteins. The empty pDEST17 vector was used as control.

The recombinant proteins were expressed in *E. coli* cells genetically engineered to produce all-*trans* lycopene by plasmid pACCRT-EIB (Misawa et al., 1995). The pACCRT-EIB *E. coli* cells display a red coloration given by the accumulation of lycopene (Figure 2). Supplementary Figure 6 shows the mass spectra obtained by LC-ESI-QTOF-MS/MS of the lycopene standard (STD) and the lycopene produced by the pACCRT-EIB *E. coli* cells, respectively. In both samples, the molecular ion of lycopene in the MS/MS spectra is observed at *m/z* 536.3, along with its M + 1 ion at *m/z* 537.3 and M + 2 ion at *m/z* 538.3 (Supplementary Figure 6A and Table 1). The lycopene molecular ion *m/z* 536.3 at 15 eV of positive ion collision is dissociated in two main ion fragments corresponding to *m/z* 444.3 [M − 92]^+^ and *m/z* 467.3 [M − 69]^+^ (Supplementary Figures 6B–D; van Breemen, 2001; Rivera et al., 2014; Amorim et al., 2018). The mass spectrum of the lycopene standard and that of lycopene extracted from pACCRT-EIB *E. coli* cells showed a similar ion pattern that corresponds to lycopene (Supplementary Figure 6 and Table 1).

**TABLE 1:** ESI-MS and MS/MS fragments of lycopene, bixin aldehyde, and norbixin reported and found in bacterial extracts that express BoCCD1 and BoCCD4 proteins.

Compound	MS (*m/z*)	MS/MS (*m/z*)
Lycopene	536 [M]^+^	536 [M]^+^
	537 [M + 1]^+^	647 [M - 69]^+^
	538 [M + 2]^+^	444 [M - 92]^+^
BoCCDs	536	536
	537	647
	538	444
Bixin aldehyde	349 [M + 1]^+^	349 [M + 1]^+^
		145
		174
		227
		265
		267
		319
BoCCDs	349 [M + 1]^+^	349 [M + 1]^+^
		145 [M + 203]^+^
		175 [M + 1 - 174]^+^
		227 [M - 121]^+^
		228 [M + 1 - 121]^+^
		264 [M - 84]^+^
		265 [M + 1 - 84]^+^
		266 [M - 82]^+^
		319 [M - 29]^+^
Norbixin	380 [M]^+^	381 [M + 1]^+^
		121
		197
		253
		267
		281
		309
BoCCDs	380 [M]^+^	380 [M]^+^
		120 [M + 1 - 261]^+^
		196 [M - 184]^+^
		252 [M - 128]^+^
		266 [M + 1 - 115]^+^
		280 [M - 100]^+^
		308 [M - 72]^+^

The color of the pellet generated after the induction of the expression of BoCCD1 and BoCCD4 proteins in pACCRT-EIB *E. coli* cells showed that only BoCCD1-1, BoCCD1-3, and BoCCD4-3 generated an evident decoloration compared to the control (empty vector). Pellet decoloration was also observed, although to a lesser extent (pellets presented a pink tone), in lycopene-producing *E. coli* cells that expressed BoCCD1-4, BoCCD4-1, and BoCCD4-2 proteins. The pellet color of *E. coli* cells that expressed BoCCD4-4 was similar to the control; both presented a red/orange color (Figure 2).

Pellet decoloration intensity is an indication that lycopene is being cleaved by the activity of the expressed CCD proteins, generating new colorless compounds (Carballo-Uicab et al., 2019; Gómez-Gómez et al., 2020; Nawade et al., 2020). Mass spectrometry analysis by direct injection of lycopene-producing *E. coli cell* extracts expressing the BoCCD1 and BoCCD4 proteins revealed a decrease in the lycopene peak (*m/z* 536.3) compared to the control, even in BoCCD4-4, which has a red orange color (Supplementary Figure 7). These results suggest a cleavage activity on lycopene, which may be related to the pellet color observed after the expression of the BoCCD proteins (Figure 2).

**FIGURE 3:**
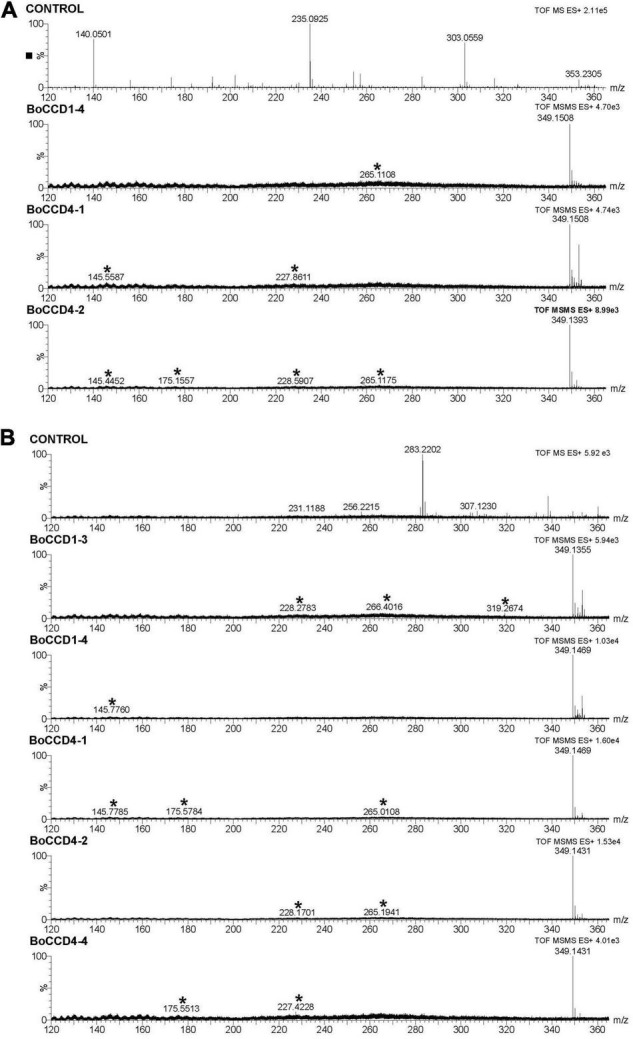
Bixin aldehyde MS/MS spectra (*m/z* 349.1) of the extracts obtained from the *in vivo* and *in vitro* expression of the BoCCD1 and BoCCD4 proteins. **(A)** MS/MS spectra of the *in vivo* expression of BoCCD1-4, BoCCD4-1 and CCD4-2. **(B)** MS/MS spectra of the *in vitro* expression of BoCCD1-3, BoCCD1-4, BoCCD4-1, BoCCD4-2, and CCD4-4. The empty pDEST17 vector was used as a control.

A previous study indicated that BoCCD1-1 and BoCCD4-3 enzymes perform lycopene cleavage at 5,6/5′,6′ bonds to produce bixin aldehyde (*m/z* 348), identified in its protonated form *m/z* 349 [M + 1] (Carballo-Uicab et al., 2019). To determine if the rest of the isolated BoCCD1 and BoCCD4 proteins are capable of producing bixin aldehyde, we performed a MS and MS/MS analysis directed at the *m/z* 349 peak. LC-ESI-QTOF-MS/MS analyzes showed that BoCCD1-1 and BoCCD4-3 produce a corresponding peak of *m/z* 349.1 (Supplementary Figure 8A), and revealed that BoCCD1-4, BoCCD4-1, and BoCCD4-2 produced the same peak of *m/z* 349.1 (Figure 3A). Unfortunately, there is still no commercial standard for bixin aldehyde. Therefore, to establish that the *m/z* 349.1 peak corresponds to bixin aldehyde we carried out its ion fragmentation. In all the bacterial extracts, the peak corresponding to *m/z* 349.1 was found, as well as in most the ion fragments of *m/z* 145, *m/z* 175 and *m/z* 265 generated by the ion fragmentation of bixin aldehyde according to the database of The Metabolomics Innovation Center (TMIC). We also found other ion fragments corresponding to bixin aldehyde according to the TMIC database (Figure 3A, Table 1, and Supplementary Figures 8A, 9). Ion fragments appear in very low intensities, even increasing the positive collision energy (> 20eV). It has been reported that diapocarotenoids or dyaldehydes produced from the oxidative cleavage of carotenoids have a low ionization efficiency in mass spectrometry (Mi et al., 2019). In the case of BoCCD1-3 and BoCCD4-4 we found a very small peak corresponding to *m/z* 350 [M + 2]^+^ and *m/z* 349 [M + 1]^+^, respectively, that was impossible to fragment (Supplementary Figure 10). In all the bacterial extracts the bixin aldehyde peak (*m/z* 349.1) was not prominent when injected directly (Supplementary Figure 7). It is also possible that the low intensities are due to the low amount of bixin aldehyde, or its high consumption efficiency (Rubio-Moraga et al., 2008; Mi et al., 2019). Results indicate that the BoCCD1-4, BoCCD4-1, and BoCCD4- 2 enzymes are capable of cleaving lycopene at 5,6/5′,6′ double bonds to produce bixin aldehyde, as BoCCD1-1 and BoCCD4-3 do.

### *In vitro* Analysis Confirms Cleavage Activity of BoCCD1 and BoCCD4 Enzymes Upon Lycopene to Produce Bixin Aldehyde

To confirm the *in vivo* cleavage activity at 5,6, 5′/6′ double bonds of lycopene, we evaluated the *in vitro* enzymatic activity of BoCCD1 and BoCCD4 proteins and their specificity to lycopene using the crude protein extracts and lycopene standard as substrate. The results of mass spectrophotometry confirmed that BoCCD1-1 and BoCCD4-3, as well as BoCCD1-4, BoCCD4-1, and BoCCD4-2, could produce the peak of *m/z* 349.1 corresponding to bixin aldehyde when they were incubated with the lycopene standard (Figure 3B and Supplementary Figure 8B). Bixin aldehyde was also identified based on its ion fragments (Figure 3B, Table 1, and Supplementary Figures 8B, 9). Interestingly, the *m/z* 349.1 peak was also found in the *in vitro* assay of BoCCD1-3 and BoCCD4-4 (Figure 3B). Diapocarotenoids detection is more efficient in the *in vitro* enzymatic assays because of their low quantity and high reactivity (Ruch et al., 2005; Ilg et al., 2014; Mi et al., 2019; Wang et al., 2019). This is probably why bixin aldehyde produced by BoCCD1-3 and BoCCD4-4 was better detected by *in vitro* assays.

### In *Escherichia coli* Cells Bixin Aldehyde Is Oxidized to Norbixin

**FIGURE 4:**
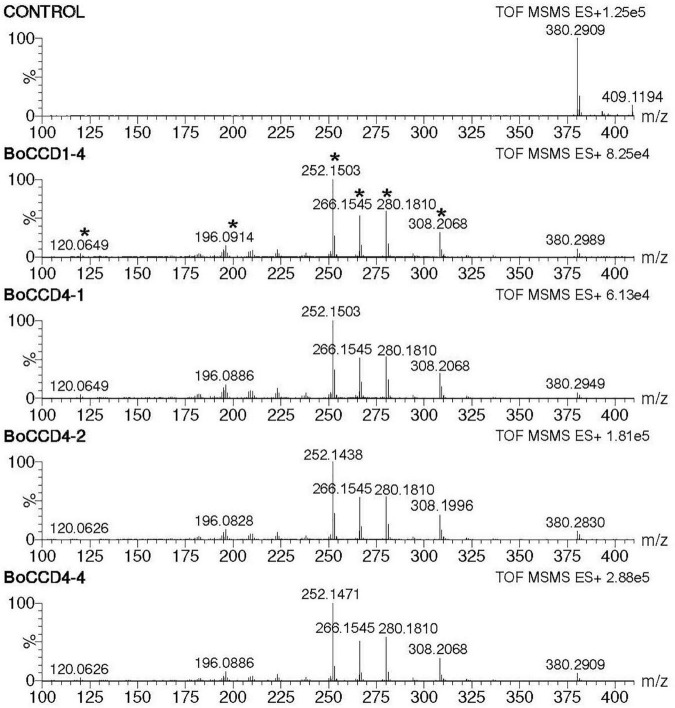
Norbixin MS/MS spectra (*m/z* 380.2) of the extracts obtained from the *in vivo* expression of BoCCD1-4, BoCCD4-1, BoCCD4-2, and CCD4-4. The empty pDEST17 vector was used as a control.

During the direct injection of the extracts obtained from the *in vivo* assay, we observed a prominent peak of *m/z* 380.2. This was observed in the extracts obtained from the *in vivo* expression of the BoCCD1 and BoCCD4 proteins (Supplementary Figure 7). This led us to suggest that *in vivo*, the aldehyde groups of bixin aldehyde are oxidized to produce norbixin whose exact mass is 380.198. To test our hypothesis, we directed the search toward the peak of *m/z* 380.2, and we analyzed whether its ionic fragmentation corresponded to norbixin (Table 1). Mass spectrophotometry results showed that only in the bacterial extracts obtained from the expression of BoCCD1-4, BoCCD4-1, BoCCD4-2, and BoCCD4-4 proteins, the peak of *m/z* 380.2 presented the ion fragmentation pattern without a positive charge that corresponds to norbixin according to the TMIC database, indicating the previous formation of bixin aldehyde (Figure 4, Table 1, and Supplementary Figure 11). Furthermore, the intensity of *m/z* 380.2 decreased after its fragmentation (Supplementary Figure 12). Although the *m/z* 380.2 peak was found in the control, it did not fragment, suggesting that it could be another compound with the same mass. The results of this analysis indicate that *in vivo*, bixin aldehyde produced by BoCCD1-4, BoCCD4-1, BoCCD4-2, and BoCCD4-4 genes is oxidized to a more stable compound— norbixin— the second product of bixin biosynthesis pathway.

**FIGURE 5:**
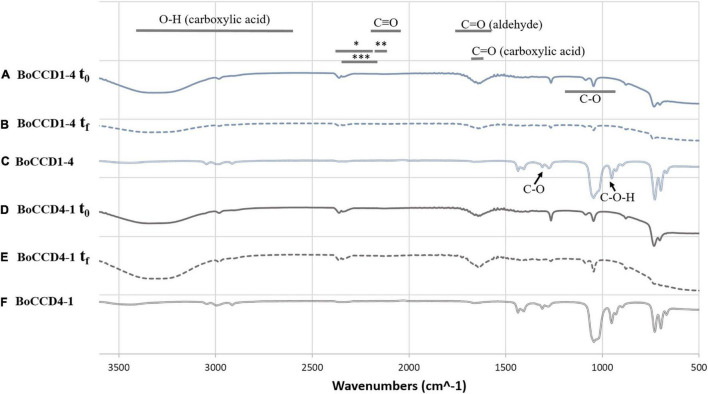
FTIR spectrum of the *in vitro* and *in vivo* extracts of BoCCD1-4 and BoCCD4-1. FTIR spectrum of the initial time (t_0_) of the *in vitro* enzymatic reaction of **(A)** BoCCD1-4 and **(D)** BoCCD4-1. FTIR spectrum of the end time (t_f_) of the *in vitro* enzymatic reaction of **(B)** BoCCD1-4 and **(E)** BoCCD4-1. FTIR spectrum of the *in vivo* assay of **(C)** BoCCD1-4 and **(F)** BoCCD4-1.

The *in vivo* formation of norbixin (which contains the carboxyl acid functional group) and *in vitro* formation of bixin aldehyde (which contains the aldehyde functional group) was corroborated by FTIR spectroscopy. The extracts of BoCCD1-4 and BoCCD4-1 were analyzed in a spectrum range from 500 to 3,600 cm^–1^ (Figure 5). The FTIR spectra within BoCCD1-4 and BoCCD4-1 in the *in vivo* and *in vitro* assays were very similar. In the initial time (t_0_) of the *in vitro* enzymatic reaction, the vibration signal of the C = O group at 1727 cm^–1^ corresponding to the aldehyde group was observed. Such signal is observed 20 h later, at the end of the *in vitro* reaction (t_f_). The vibration signal at 2250 cm^–1^ is also observed in both t_0_ and t_f_, attributed to the different products generated by the changing bonds of the CHO (*), CH (^**^) and CH3 (^***^) groups until bixin aldehyde is produced from lycopene. The C triple bond signal is also observed at 2100 to 2250 cm^–1^ (Figures 5A,B,D,E; Carballo-Uicab et al., 2019). On the other hand, in the *in vivo* assay of BoCCD1-4 and BoCCD4-4, the vibration signal of the C = O group at 1727 cm^–1^ corresponding to the aldehyde group, and of the CHO (*), CH (^**^) and CH3 (^***^) groups between 2100 cm^–1^ to 2250 cm^–1^ is almost imperceptible. Instead, the vibration signal of the C-O-H group at 935 cm^–1^, which corresponds to the carboxyl acid group is observed. We also observed the vibration signals of the C-O at 1294 cm^–1^, C = O at 1714 cm^–1^, and the OH stretch between 3,400 to 2,400 cm^–1^, characteristic of compounds with carboxyl acid groups (Figures 5C,F). The FTIR spectra of the *in vivo* and *in vitro* assays corroborate the mass spectrometry results. It also suggests that the formation of bixin aldehyde occurs by a rapid enzymatic reaction because the C = O vibration signal is observed since t_0_ in the *in vitro* enzymatic reaction (Figures 5A,D). It is also possible that the *in vivo* formation of norbixin from bixin aldehyde occurs continuously, which could explain why the bixin aldehyde signal was almost imperceptible in these samples.

### Structural 3D Modeling and Docking Analysis of BoCCD1 and BoCCD4 Proteins

**FIGURE 6:**
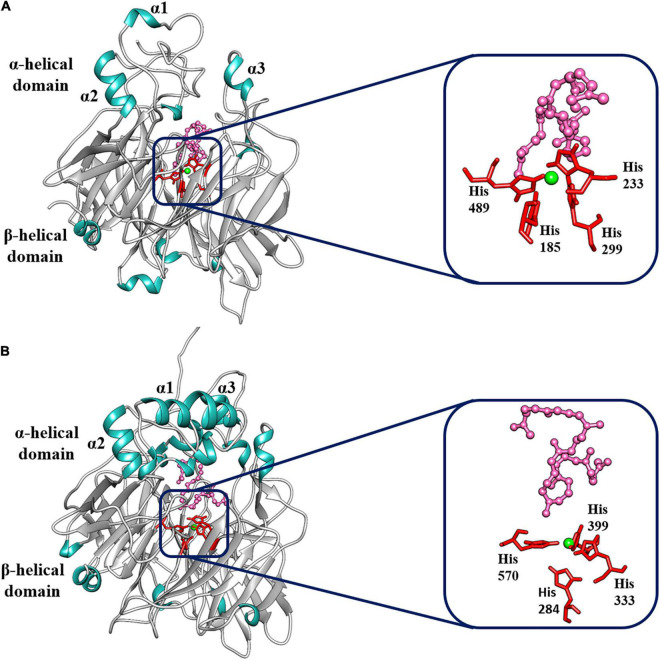
Best-fit 3D model and docking analysis of **(A)** BoCCD1-4 and **(B)** BoCCD4-2 proteins. The α-helix (α1, α2, and α3) and the β-propeller domains are indicated on the models. Green shows the Fe^2+^ ion coordinated by the four histidine residues. The four histidine residues are shown in red, and the lycopene molecule is shown in pink.

To identify the structural 3D domains of the BoCCD1 and BoCCD4 proteins, structural modeling of sequence homology was performed. Sequence alignment software predicted VP14 — a 9-Z-epoxycarotenoid dioxygenase from maize (PBD accession: 3npeA) — as the best template to model the different BoCCDs proteins (Schwartz et al., 1997; Messing et al., 2010). In all cases, we found reasonable identity and confidence percentages between VP14 and the BoCCD1 and BoCCD4 proteins, indicating good coverage between the residues (Supplementary Table 4). The structural models obtained for BoCCD1-1, BoCCD1-3, BoCC1-4, BoCCD4-1, BoCCD4-2, BoCCD4-3, and BoCCD4-4 proteins using the VP14 protein as a template showed that all models obeyed the basic conformation of the CCD enzymes (Figure 6 and Supplementary Figure 13). In general, the seven CCDs contain the two main functional domains exhibiting the seven characteristic blades that form the β-propeller domain, in addition to the three α-helices (α1, α2, and α3) that form the α helical domain located in the upper part of the β-propeller. The β-propeller domain is conserved in the CCD protein family, it forms a toroidal structure with a long tunnel in the central axis surrounded by the seven blades. The four histidine residues coordinated with Fe^2+^ necessary for dioxygenase activity are in the center of the structure (Figure 6 and Supplementary Figure 13; Kloer and Schulz, 2006; Messing et al., 2010; Harrison and Bugg, 2014; Priya et al., 2017; Vega-Teijido et al., 2019; Daruwalla and Kiser, 2020). In VP14, the parallel helices α1 and α3 form a hydrophobic patch that is associated with the interaction of this protein with the thylakoid membranes (Tan et al., 2001; Messing et al., 2010; Daruwalla and Kiser, 2020).

To gain a better understanding of the molecular mechanism of BoCCD1 and BoCCD4, a docking analysis was directed toward the center of the BoCCD1 and BoCCD4 proteins where Fe^2+^ is located, using lycopene as a ligand. Results showed that all BoCCD1 and BoCCD4 proteins were able to form molecular interactions with lycopene. The spatial location of the iron (II) ion coordinated with the four conserved histidine residues were very similar in all BoCCDs. The only significant differences were found in the spatial orientation of lycopene, which probably influenced the differential residues interacting with it (Figure 6 and Supplementary Figure 13). The lowest RMSD values for all BoCCDs indicate the accuracy of the docking geometries and suggest the high conservation of the binding site structure of all BoCCD1 and BoCCD4 proteins (Supplementary Table 5). Differences were found in their binding affinities estimated by ΔG° values and dissociation constants (Kd). The range of ΔG° values is between −5 and −7 in all BoCCDS, and it is reasonable assuming that the same ligand was used. The best affinity value is for CCD4-2, followed by BoCCD1-1, BoCCD4-1, BoCCD4-4, BoCCD1-3, BoCCD1-4, and finally BoCCD4-3 (Supplementary Table 5). The low Kd values also suggest that CCD4-2 and CCD1-1 may be playing a more relevant role in the formation of bixin aldehyde from lycopene. The results found in the *in silico* analysis indicate that all BoCCD1 and BoCCD4 proteins contain the structural and functional characteristics of carotenoid cleavage dioxygenases and confirm that lycopene can be used as a substrate to produce bixin aldehyde.

## Discussion

### Bixin Aldehyde Biosynthesis Is Performed by Different Members of the BoCCD1 and BoCCD4 Subfamily of Enzymes

Bixin biosynthesis pathway begins with the biosynthesis of bixin aldehyde through the symmetric cleavage of lycopene at 5, 6/5′,6′ double bonds by a lycopene dioxygenase (BoLCD) (Jako et al., 2002; Bouvier et al., 2003; Carballo-Uicab et al., 2019). The transcriptomes of leaf and mature/immature seed of a local Yucatecan variety of *B. orellana* allowed the identification of a new set of eight candidate *BoCCD* genes potentially involved in bixin biosynthesis. These genes were grouped into two subfamilies, four belong to the *BoCCD1* subfamily: *BoCCD1-1*, *BoCCD1-2*, *BoCCD1-3*, and *BoCCD1-4*; and four to the *BoCCD4* subfamily: *BoCCD4-1*, *BoCCD4-2*, *BoCCD4-3*, and *BoCCD4-4* (Cárdenas-Conejo et al., 2015). Until now, the biosynthesis of bixin aldehyde from lycopene had only been demonstrated for BoCCD1-1 and BoCCD4-3. However, based on previous research results, we hypothesized that the rest of the BoCCD1 and BoCCD4 proteins could have a possible role in bixin biosynthesis (Cárdenas-Conejo et al., 2015; Carballo-Uicab et al., 2019). Therefore, the main objective of this work was to determine if the proteins encoded by *BoCCD1-3*, *BoCCD1-4*, *BoCCD4-1*, *BoCCD4-2*, and *BoCCD4-4* genes (in addition to *BoCCD1-1* and *BoCCD4-3*) could produce bixin aldehyde.

The results of the analysis of the deduced amino acid sequence of the isolated *BoCCD1* (*BoCCD1-1*, *BoCCD1-3*, *BoCCD1-4*), and *BoCCD4* (*BoCCD4-1*, *BoCCD4-2*, *BoCCD4-3*, and *BoCCD4-4*) genes indicated that they were the same genes identified in the *B. orellana* transcriptome (similarity > 98%) (Supplementary Table 2; Cárdenas-Conejo et al., 2015). Sequence alignment also showed that they all contain the four conserved histidine residues of the active site as well as the glutamate and aspartate residues necessary for dioxygenase activity (Supplementary Figure 4; Harrison and Bugg, 2014; Daruwalla and Kiser, 2020). Additionally, 3D modeling showed that all BoCCD1 and BoCCD4 proteins obey the basic conformation of CCD enzymes, contain the hydrophobic α hélix domain predicted for the interaction with plastid membranes and the β-propeller domain with the catalytic site in the center of their structure, indicating that they encode functional carotene cleavage dioxygenases (Figure 6 and Supplementary Figure 13; Tan et al., 2001; Messing et al., 2010; Daruwalla and Kiser, 2020).

The functional activity of the BoCCD1 and BoCCD4 proteins in the production of bixin aldehyde from lycopene was evaluated using *in vivo* (*E. coli*) and *in vitro* approaches. The results of the mass spectrometry analysis of the *in vivo* and *in vitro* assays showed that the BoCCD1-3, BoCCD1-4, BoCCD4-1, BoCCD4-2, and BoCDD4-4 proteins also cleaved lycopene at the 5,6/5′,6′ double bonds producing bixin aldehyde (*m/z* 349). Identification was based on ion fragmentation (Figure 3, Table 1, and Supplementary Figure 9). These results are consistent with the ΔG° values from the docking analysis, which suggest that all BoCCD1 and BoCCD4 proteins can use lycopene as a substrate (Supplementary Table 5). In some extracts from the *in vivo* assay, bixin aldehyde was not detected as a prominent peak. Something similar has been observed in other works carried out in *E. coli*, where the dialdehydes were not detected in appreciable amounts (Schwartz et al., 2001; Vogel et al., 2008). It has been reported that diapocarotenoids or dialdehydes can be detected more efficiently in the *in vitro* assays, because they are generally found in low quantities and are rapidly catabolized (Ruch et al., 2005; Ilg et al., 2014; Mi et al., 2019; Wang et al., 2019).

### In *Escherichia coli* Cells, Bixin Aldehyde Can Be Oxidized to Norbixin, the Second Compound of the Bixin Biosynthesis Pathway

Interestingly, we found that in the *in vivo* assay of BoCCD1-4, BoCCD4-1, BoCCD-4-2, and BoCCD4-4, bixin aldehyde was oxidized to norbixin (*m/z* 380), the second product of the bixin biosynthesis pathway (Figure 4). Norbixin was also identified by its ion fragmentation (Supplementary Figure 11 and Table 1). One line of evidence that norbixin is being produced by BoCCD1-4, BoCCD4-1, BoCCD4-2, and BoCCD4-4 is the red/orange color of the pellets observed after their expressions. Norbixin has a natural red/orange to yellow color, depending on its concentration (Sharma et al., 2020). We suggest that *E. coli* must contain a non-specific aldehyde dehydrogenase capable of oxidizing bixin aldehyde produced by the BoCCD enzymes to norbixin. In *E. coli* cells, crocetin dialdehyde produced by the cleavage activity of CCD2 at the 7,8/7′,8′ double bonds of zeaxanthin is oxidized to crocetin by an unknown aldehyde dehydrogenase (Frusciante et al., 2014). A similar result was found by Jimenez-Lopez et al. (2016), reinforcing the idea of an *E. coli* aldehyde dehydrogenase capable of oxidizing the cleaved aldehyde product. Moreover, the signal vibration of the aldehyde group of bixin aldehyde and of the carboxyl acid groups of norbixin were detected in the extracts of the *in vivo* and *in vitro* assay of the BoCCDs (Figure 5).

To estimate the amount of lycopene cleaved and the amount of bixin aldehyde and norbixin produced, we analyzed the intensity level of the molecular ion of each compound. The values found suggest that in the *in vivo* assay: a) BoCCD1-1, BoCCD4-2, and BoCCD4-4 cleave lycopene more efficiently; b) BoCCD1-1, BoCCD4-3, and BoCCD4-2 produce more bixin aldehyde; and c) bixin aldehyde is more efficiently oxidized to norbixin by BoCCD4-4, followed by BoCCD4-2 (Supplementary Table 6). Even norbixin was only detected in the *in vitro* BoCCD4-4 assay (Supplementary Figure 14). The high production of bixin aldehyde by BoCCD1-1 and BoCCD4-2 is also related to the low values of the dissociation constant (Kd) toward lycopene of these enzymes (Supplementary Table 5). Although in the *in vitro* assay we used equal amounts of synthetic lycopene, we did not observe as obvious changes in coloration as in the *in vivo* assay; for this reason, we considered the values found in the *in vivo* assays, where the activity of BoCCD1 and BoCCD4 is more efficient. The activity of CCD enzymes can be greatly reduced during protein extraction and purification (Rodríguez-Concepción and Welsch, 2020).

### BoCCD1 and CCD4 Enzymes Could Act in a Coordinated Way for the Biosynthesis of Bixin and Other Apocarotenoids in *Bixa orellana* Plants

*Bixa orellana* produces and accumulates bixin mainly in its seeds, but bixin has also been detected in other plant tissues such as roots, leaves, stems, floral buds, and flowers (Mahendranath et al., 2011; Rodríguez-Ávila et al., 2011a,b). We propose that *in planta*, all BoCCD1 and BoCCD4 enzymes participate in bixin biosynthesis in a tissue-specific manner. The presence of families of enzymes that perform the same enzymatic activity in the biosynthesis of carotenoids and apocarotenoids seems to be common, allowing the biosynthesis of these compounds to be tissue-specific and specific to a stage of development (Rubio-Moraga et al., 2008; Walter and Strack, 2011; Cárdenas-Conejo et al., 2015). Expression analyzes of *CCD1* and *CCD4* genes from different plant species show that they individually have tissue-specific expressions and that their expression is often regulated by the accumulation of carotenoids and apocarotenoids (Simkin et al., 2004; Rubio-Moraga et al., 2008, 2014; Rodríguez-Ávila et al., 2011b; Frusciante et al., 2014). *BoCCD1*, orthologous of *BoCCD1-1*, was expressed in root, stem, leaves, floral tissues, and seeds of *B. orellana*, but its expression was only highly induced in immature seeds when pigment production began (Rodríguez-Ávila et al., 2011b; Cárdenas-Conejo et al., 2015). Carballo-Uicab et al. (2019) also found that the expression of BoCCD1-1 and BoCCD4-3 was markedly high in bixin storage cells (BSCs) of the arils of *B. orellana* seeds, coinciding with the accumulation of bixin. In leaves of *B. orellana*, a positive correlation was observed between the content of bixin and the expression of the *BoCCD4-3* and *BoCCD4-2* genes (Faria et al., 2020). This suggests that more than one BoCCD enzyme can carry out bixin biosynthesis in the same tissue. According to the prediction of the subcellular location and our findings, bixin biosynthesis can be performed both in the cytosol and in plastids, probably by independent pathways, but acting in coordination. The prediction of the subcellular location of the *BoALDH* and *BoSABATH* gene families showed that several members can be located in the cytosol or plastids, supporting this proposal (Cárdenas-Conejo et al., 2015). BoCCD1 proteins probably anchor to plastid membranes to gain access to the lycopene, as predictions of the subcellular location indicated that they are cytosolic (Floss and Walter, 2009; Frusciante et al., 2014). Conversely, BoCCD4 proteins have a plastid target sequence, which would give them direct access to lycopene (Rubio-Moraga et al., 2008; Vranová et al., 2013).

Cell decoloration after the expression of CCD enzymes in *E. coli* cells that produce pigmented carotenoids indicates that they are metabolized to new cleaved colorless compounds (Schwartz et al., 2001; Huang et al., 2009; Nawade et al., 2020). In BoCCD1-1, BoCCD1-3, and BoCCD4-3, we observed a decolored pellet after its expression in lycopene-producing *E. coli* cells. These enzymes are likely to cleave lycopene or its precursors at other bonds, generating mostly volatile compounds that were not detectable by LC-ESI-QTOF-MS/MS. CCD1 enzymes can cleave lycopene at the 5, 6 or 5′, 6′, 9, 10 or 9′, 10′, and 7, 8 or 7′, 8′ bonds, producing the volatile MHO (6-methyl-5-hepten-2-one), pseudoionone, and geranial, respectively (Vogel et al., 2008; Huang et al., 2009; Ilg et al., 2009; Lashbrooke et al., 2013; Nawade et al., 2020). CCD4 enzymes can also produce MHO and cleave lycopene at 7, 8 and 5, 6 bonds to produce apo-8′-lycopenal (C_30_), and apo-10′-lycopenal (C_27_), respectively (Lashbrooke et al., 2013; Rodrigo et al., 2013). Moreover, CCD1 and CCD4 can cleave C_30_ and C_27_ apolycopenals at various bonds producing pseudoionone and C_15_, C_17_, C_19_, C_20_, C_22_, and C_25_ dialdehydes (Ilg et al., 2009; Rodrigo et al., 2013). CCD1 and CCD4 enzymes can also cleave lycopene precursors such as phytoene and ζ-carotene producing geranylacetone and farnesyl acetone (Rubio-Moraga et al., 2008; Vogel et al., 2008; Lashbrooke et al., 2013; Ilg et al., 2014). The volatile apocarotenoids produced by the CCD1 and CCD4 enzymes are responsible for flavor, aroma, pollinators attraction, and signaling (Floss et al., 2008; Rubio-Moraga et al., 2008; Floss and Walter, 2009; Walter et al., 2010; Brandi et al., 2011). A variety of linear and cyclic apocarotenoids have been found in *B. orellana*, as well as volatile aldehyde, ketones, esters, and carboxylic acids compounds derived from the polyene chain, whose biosynthesis is still unknown (Mercadante et al., 1996, 1997; Galindo-Cuspinera et al., 2002; de Oliveira Júnior et al., 2019). The presence of multiple BoCCD1 and BoCCD4 enzymes in *B. orellana* opens the possibility of CCDs having different substrate specificity, although further studies are needed to confirm this. CCD1 enzymes are considered more promiscuous due to their wide substrate preference, while CCD4 enzymes appear to be more substrate specific (Vogel et al., 2008; Huang et al., 2009; Walter and Strack, 2011).

The elucidation of the enzymes involved in the bixin biosynthesis pathway, as well as their regulatory mechanisms are essential to improve the production of this important apocarotenoid. Although the main objective of this work was to find the BoCCD1 and BoCCD4 enzymes involved in the first step of the bixin biosynthesis pathway, in this work, it was also possible to shed light on the second step of the bixin biosynthesis pathway that had not been evaluated for a long time. *B. orellana* contains several potential members of the ALDH family of enzymes that can carry out the oxidation of bixin aldehyde; thus future work will aim at elucidating the BoALDH enzymes responsible for norbixin biosynthesis. It is also possible that the BoCCD1 and BoCCD4 enzymes carry out bixin biosynthesis in a tissue-specific way, and that they could be involved in other biological processes through the biosynthesis of other apocarotenoids by cleaving different carotenoids. Although further biochemical and molecular studies are needed, the difference in the production of bixin aldehyde and norbixin seems to support our argument. Finally, the elucidation of methyltransferases (BoSABATHS) that catalyze the last step of bixin biosynthesis opens the possibility of scaling the industrial production of bixin in heterologous organisms such as bacteria and plants.

Recent studies carried out on *Nicothiana tabacum* and *Solanum lycopersicum* show plants as potential alternatives for the production of apocarotenoids of commercial interest such as crocin through the expression of a carotenoid-cleaving dioxygenase enzyme from *C. sativus* (CsCCD2) and *B. orellana* (BoCCD4-3), respectively (Martí et al., 2020; Frusciante et al., 2021). Performing metabolic engineering with the BoCCD1s and BoCCD4s identified in this work in plants such as *S. lycopersicum* and *Carica papaya*, which contain different carotenoids in addition to lycopene, would allow evaluating them as possible alternatives for the production of bixin and probably other apocarotenoids, given the presence of different carotenoids in these plants and the ability of CCD1 and CCD4 enzymes to cleave them at different double bond sites.

## Data Availability Statement

The datasets presented in this study can be found in online repositories. The names of the repository/repositories and accession number(s) can be found below: Protein accession numbers (10.6084/m9.figshare.18461612), FTIR data (10.6084/m9.figshare.18480581), and Mass spectrometry data (10.6084/m9.figshare.18482666).

## Author Contributions

RU-C conducted most of the experiments, conceived and designed the experiments, and drafted the manuscript. MA-E helped in data collection, pigment extraction, and sample preparation. JR-C helped in data collection and establishment of mass spectrometry methodology. AV-C helped in data collection, mass spectrometry, and FTIR spectroscopy analysis. VC-U helped in 3D structural modeling and docking analysis. HS-P helped in the supervision of 3D structural modeling and docking analysis. RR-M coordinated the project, conceived the experiments, and reviewed and edited the manuscript. All authors contributed to the article and approved the submitted version.

## Conflict of Interest

The authors declare that the research was conducted in the absence of any commercial or financial relationships that could be construed as a potential conflict of interest.

## Publisher’s Note

All claims expressed in this article are solely those of the authors and do not necessarily represent those of their affiliated organizations, or those of the publisher, the editors and the reviewers. Any product that may be evaluated in this article, or claim that may be made by its manufacturer, is not guaranteed or endorsed by the publisher.

## Footnotes

1
https://www.ncbi.nlm.nih.gov/

2
https://www.ebi.ac.uk/Tools/msa/clustalo/

3
https://web.expasy.org/translate/

4
http://ipsort.hgc.jp/

5
http://linux1.softberry.com/all.htm

6
https://urgi.versailles.inra.fr/predotar/

7
https://solgenomics.net/

8
http://www.sbg.bio.ic.ac.uk/phyre2/html/page.cgi?id=index

9
http://zinc.docking.org/

10
http://www.yasara.org/minimizationserver.htm
