# Supplementary figures and images for: Identifying Bixa orellana L. New Carotenoid Cleavage Dioxygenases 1 and 4 Potentially Involved in Bixin Biosynthesis
Source: Front Plant Sci. 2022 Feb 11;13:829089. doi: 10.3389/fpls.2022.829089 (PMC8874276; doi:10.3389/fpls.2022.829089)

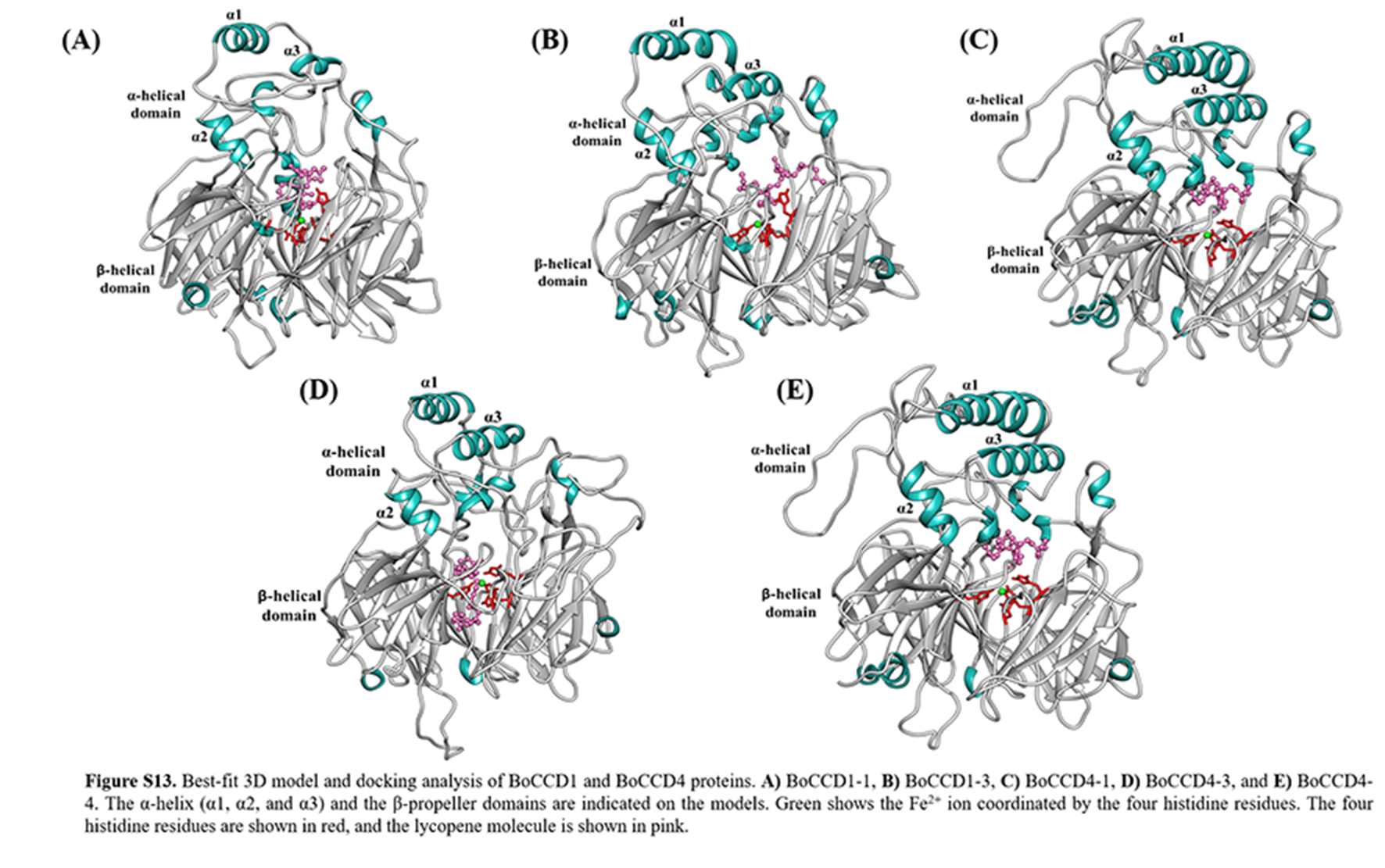

Supplement: Supplementary file 1 [file Image_1.TIF]
