# Supplementary material for: Identifying Bixa orellana L. New Carotenoid Cleavage Dioxygenases 1 and 4 Potentially Involved in Bixin Biosynthesis
Source: Front Plant Sci. 2022 Feb 11;13:829089. doi: 10.3389/fpls.2022.829089 (PMC8874276; doi:10.3389/fpls.2022.829089)
Supplement: Supplementary file 2 [file Table_1.DOCX]

**Supplementary tables**

| **Gene** | **Primer sequence** | **Tm (°C)** |
| --- | --- | --- |
| *BoCCD1-1* | CCD1-1F: TTACTCGAGATGGCTCAGGAGGCGGAGAAGC | 61 |
|  | CCD1-1R: TACTCGAGGTCTTGCCTCAAGATCTCTCCATGTG |  |
| *BoCCD1-3* | CCD1-3 F: ACACATATGGCAGAGGAGGGGAAGC | 53 |
|  | CCD1-3R: ATAGGATCCTGCCTCAGGATCTCTGCGTAC |  |
| *BoCCD1-4* | CCD1-4F: TGGGTGGAGCACCTCACCGTCAAG | 53.8 |
|  | CCD1-4R: TCTTGCCTCGGATCCCTGCCCTGC |  |
| *BoCCD4-1* | CCD4-1F: CATCATATGTTCTTCCTTCGCATGATG | 57.3 |
|  | CCD4-1R:AAAGGATCCGTGACTAACATAGGAAATCTCC |  |
| *BoCCD4-2* | CCD4-2F: CATCATATGTATTACTCATCAATCCCATTGCC | 58 |
|  | CCD4-2R: AAACTCGAGTCATAAGGACGAAATGTCCTCTG |  |
| *BoCCD4-3* | CCD4-3F: TCACAGAAGATGTACTGCTATTCCA | 58.8 |
|  | CCD4-3R: TAGTCAAGGAGTATGCACACGA |  |
| *BoCCD4-4* | CCD4-4F: CATCATATGTGCAACTTGATTTGC | 56.1 |
|  | CCD4-4R: AAAGGATCCTCATAAAGAAGAAAGATCCTCTC |  |

**Table S1.** Primer sequences used for the amplification and subsequent cloning of the coding regions of *BoCCD1*- and *BoCCD4* genes.

| **Gene** | **Nucleotides** | **% Identity** | **Amino acids** | **% Similarity** |
| --- | --- | --- | --- | --- |
| *BoCCD1-1* | 1629 | 99.51 | 542 | 99.45 |
| *BoCCD1-3* | 1644 | 99.09 | 547 | 98.54 |
| *BoCCD1-4* | 1515 | 99.93 | 504 | 99.80 |
| *BoCCD4-1* | 1800 | 98.72 | 599 | 98.00 |
| *BoCCD4-2* | 1749 | 99.37 | 582 | 99.14 |
| *BoCCD4-3* | 1773 | 98.76 | 590 | 97.11 |
| *BoCCD4-4* | 1863 | 99.73 | 620 | 99.35 |

**Table S2.** Percentage of identity and similarity of the coding regions of the isolated *BoCCD1* and *BoCCD4* genes with those reported in the *B. orellana* transcriptome.

| **Protein** | **iPSORT** | | **ProtComp 9.0** | | **PREDATOR** |
| --- | --- | --- | --- | --- | --- |
| BoCCD1-1 | | Cyt | | Cyt | Cyt |
| BoCCD1-2 | | Cyt | | Cyt | Cyt |
| BoCCD1-3 | | Cyt | | Cyt | Cyt |
| BoCCD1-4 | | Chl | | Cyt | Cyt |
| BoCCD4-1 | | Chl | | Chl | Chl |
| BoCCD4-2 | | Chl | | Chl | Chl |
| BoCCD4-3 | | Mit/Chl | | Chl | Chl |
| BoCCD4-4 | | Mit/Chl | | Chl | RE |

**Table S3.** Prediction of the subcellular location of the BoCCD1 and BoCCD4 proteins.

| Protein | Template | % Identity | % Confidence  (overlapping residues/ total residues) x 100 |
| --- | --- | --- | --- |
| BoCCD1-1 | 3npea (VP14) | 38 | 96% |
| BoCCD1-3 | 3npea (VP14) | 39 | 94% |
| BoCCD1-4 | 3npea (VP14) | 39 | 100% |
| BoCCD4-1 | 3npea (VP14) | 38 | 86% |
| BoCCD4-2 | 3npea (VP14) | 38 | 84% |
| BoCCD4-3 | 3npea (VP14) | 38 | 87% |
| BoCCD4-4 | 3npea (VP14) | 36 | 91% |

**Table S4.** Structural comparation between the VP14 template and the BoCCD1 and BoCCD4 protein models.

| Protein | ∆G° (Kcal/mol) | RMSD (Root-Mean-Square  Deviation)  (Å) | Kd  (µM) |
| --- | --- | --- | --- |
| BoCCD1-1 | -6.79 | 0 | 10.62 |
| BoCCD1-3 | -5.86 | 0 | 50.59 |
| BoCCD1-4 | -5.64 | 0 | 73.35 |
| BoCCD4-1 | -6.46 | 0 | 18.42 |
| BoCCD4-2 | -6.89 | 0 | 8.83 |
| BoCCD4-3 | -5.37 | 0 | 116.5 |
| BoCCD4-4 | -6.3 | 0 | 24.22 |

**Table S5.** Free energies of protein-ligand affinity for the best geometries obtained from the docking of lycopene to BoCCD1 and BoCCD4 proteins.

| **Sample** | **Lycopene ion intensity** | **Bixin aldehyde ion intensity** | **Norbixin ion intensity** |
| --- | --- | --- | --- |
| Control | 1.78e3 | nd | nd |
| Control | 9.71e3 | nd | nd |
| BoCCD1-1 | 232 | 1.13e4 | nd |
| BoCCD1-1 | 143 | 9.26e3 | nd |
| BoCCD1-3 | 2.37e3 | nd | nd |
| BoCCD1-3 | 242 | nd | nd |
| BoCCD1-4 | 3.07e3 | 4.70e3 | 2.50e3 |
| BoCCD1-4 | 2.01e3 | 3.51e3 | 1.77e3 |
| BoCCD4-1 | 2.88e3 | 6.72e3 | 1.17e4 |
| BoCCD4-1 | 2.15e3 | 4.74e3 | 2.73e5 |
| BoCCD4-2 | 266 | 1.82e4 | 8.15e5 |
| BoCCD4-2 | 523 | 8.99e3 | 4.33e5 |
| BoCCD4-3 | 1.17e3 | 3.74e4 | nd |
| BoCCD4-3 | 5.46e3 | 1.20e4 | nd |
| BoCCD4-4 | 236 | nd | 6.77e6 |
| BoCCD4-4 | 945 | nd | 1.61e6 |

**Table S6.** Intensity values of lycopene, bixin aldehyde, and norbixin ions in two biological replicates of the *in vivo* assay of BoCCD1 and BoCCD4 enzymes. Nd, not detected.
