# Supplementary material for: Identifying Bixa orellana L. New Carotenoid Cleavage Dioxygenases 1 and 4 Potentially Involved in Bixin Biosynthesis
Source: Front Plant Sci. 2022 Feb 11;13:829089. doi: 10.3389/fpls.2022.829089 (PMC8874276; doi:10.3389/fpls.2022.829089)
Supplement: Supplementary file 3 [file Data_Sheet_1.PDF]

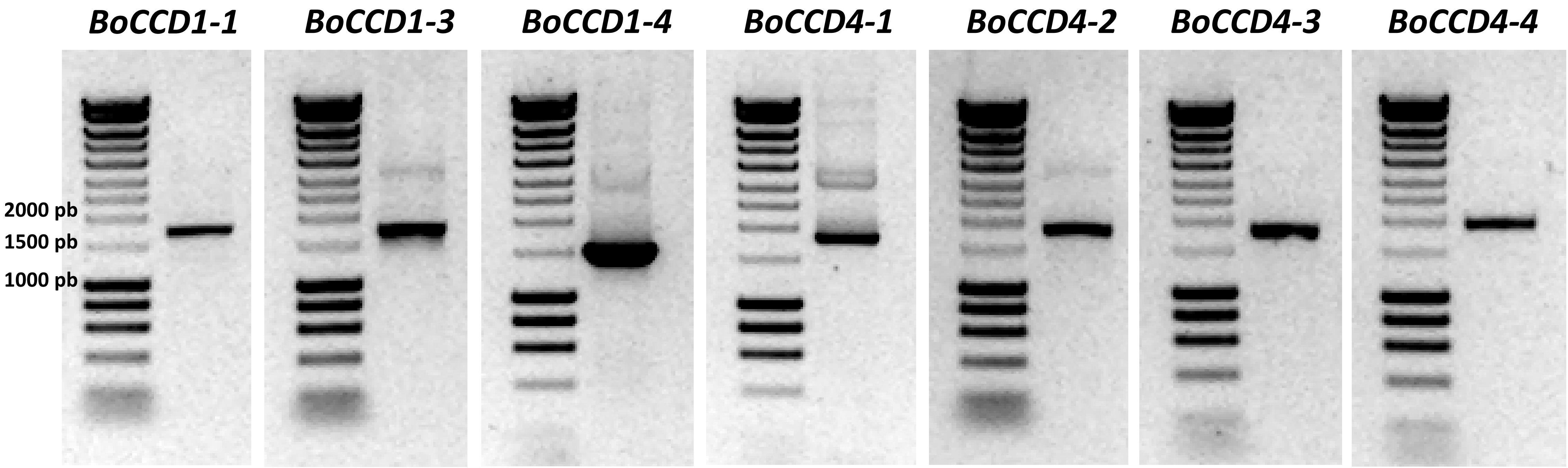

Figure S1. PCR fragment of the coding regions of the *BoCCD1* and *BoCCD4* genes amplified from *B. orellana* cDNA. The expected theoretical sizes are 1629 pb for *BoCCD1-1*, 1644 pb for *BoCCD1-3*, 1515 pb for *BoCCD1-4*, 1800 pb for *BoCCD4-1*, 1749 pb for *BoCCD4-2*, 1773 pb for *BoCCD4-3*, and 1863 pb for *BoCCD4-4*. Hyperladder I was used as molecular marker.
