# Supplementary material for: Identifying Bixa orellana L. New Carotenoid Cleavage Dioxygenases 1 and 4 Potentially Involved in Bixin Biosynthesis
Source: Front Plant Sci. 2022 Feb 11;13:829089. doi: 10.3389/fpls.2022.829089 (PMC8874276; doi:10.3389/fpls.2022.829089)
Supplement: Supplementary file 4 [file Data_Sheet_2.PDF]

BoCCD1-1

MAQEA**G**KQAPRGGPRNGILEVNPKPQKGLKSTLIDWLEKLIVKLMHDPSQPLPFLTGNFAPIPHETPPTTDLPVKGHLPECLNGEFVRVGPNPKFAPVAGYH
WFDGDGMVHGMRIKDGKAAYVSRYVNTSRFKQEDFFEGPKFMKIGDLKGLFGLLMVHLQILRAKTKVIDTTYGVGTGNTALVYHHGKLLALNEGDKPY
VLKVLEDGDLQTIGLLDYDKRLSHSFTAHPKVDPVTGEMFTFGYSHEAPYITYRVISKDGFMHDPVPITISEPIMMHDFAITENYAIFLDLPLHFRPKEMVKE
KKMIFSFDPTKKARFGVLPRYAKDDLLIRWFELPNCFIFHNANAWEEGDDVVLITCRIEDPDLDNKKLGSFKNE**L**YEMRFNMKTGEASEKQLSASAVDFPR
VNESYTGRKQRYVYGTILDEKAKVTGIAKFDLHAEPEPGKARIEVGGNVKGVFDLGAGRYGSEAVFVPREPGTTSDEDDGYLIFFLHDENTGKSFVNVIDA
KTMSADPVAVVELPHRVPYGFHAFFVTEEQLQEQAKL

BoCCD1-3

MAEEGKQAYGGGARKGLVEINPKPQKGLASRAIDWLEKLIVKLMYDSSHPLYYLTGNYAPLPRETPPTKDLPVQGFLPECLNGEFVRIGPNPKFHPVAGYH
WFDGDGMVHGL**RV**KDGKATYVSRFVRT**S**RLKQEEFHGGAKFW**K**LGDLKGIFG**L**LMVSIGLLRAKTKVLDLSYGDGSGNTALVYHQGKLLALHEQDKPY
VLQVLEDGDLQTLGLLDYDKRLSHAFTAHPKVDPVTDEMFTFGYSSTPPYITYRVISKDGFM**R**DPVPITVSRAIMMHDFAITENYAIFMDLPLYFDIKEMIKG
GRHMVSFDETKKARFGVLDRYAKDDLLIKWFELPNCYIFHNANAWEEGDEVVLVSCRIDKIDFDVFDEPVKKKLG**N**LQNELYEMRFNMKTGLATQKKLS
ESSLDFPRVNDCYTGRKTRYVYATNIDNFAKVKRIVKFDLNAEPEPGKTCIELGGNVKGIYDLGPGRFGSEAVFVPREPGRNFEEDDGYLILFVHDENVGKS
FVNVVDAKTMSSDPIAVIELPNRVPYGFHAFFVPEEQLKAQARF

BoCCD1-4

MHHSSGPPHFLSGNFAPVSQETPPTKNLSVRGYLPECLNGEFVRIGPNPKFSPLSGYHWFDGDGMVHGLRIKDGKATYVSRYVRTSRLNQEEFYGGARFRK
VGDFKGLFGILMVIYEVLREKTKVLDFSYGLGTGNTALAYHHGKLLALNERDKPYVLKVLEDGDLQTLGMLDYDKRLSHTFTAHPKVDPITGEMFTFGYS
RMPPYITYRVISKDGVMHDPVPITISQPIMMHDFAITEKYAIFMDLPLYFKPEEMMKQKKRMFPFDPTKQARFGILARYAKDELLIRWFELPNCYIFHTANA
WEEGDEVVLIVCRIENMDLDAYEEPAKKELENFRTELYEMRFNMKTGLASQKKLSAPSVEFPRVNEC**Y**IGRKQRYVYAANMDESAKIKRIVKFDLNAEPEP
GKTSIEVGGNVRGIFDLGSGIFGSEAVFVPREPGSNLEEDDGYLIVFAHDENAGKSFVNVIDARTMSSDSIAVVELPSRVPYGFHGLFVPEEQLEEQAKF

BoCCD4-1

MMYYSISSSFRIDTICYHDNKKYSKFSNKQAGESRLHHPKKSFSF**S**IKPNLHFQKLKMGMSQQKITQNFPPPP**SF**LPHLASMSFLQIIFSSLSKLIAPPLDLWIDP
SHVFTENFAPVEEMDPIECPIEGELPPSLHGAAYIRNGTNPQYKPQRALHIFEGDGMLHSLRFSEGDRAVYSSRYVRTYKFMIERGRGAAIIPTFFSGFYGLIDI
ASVFT**F**LWELVRSRVNPMNHGFGGANVSLGLL**A**KKLLALNESDLPYIMNLTDDGDIETVGRWDFDEGLLASMTAHPKFDKDTKETFAFRYLSLLHPYLTFF
KFDQNGVKQNEVKISSMERLCFIHDFAVTKRFLIIEETQLAASIAKVLLGRGSMFYYNPKNTPRFGVLPRHATDESELMWFQAPGFNAMHYINAWENEEDD
DEIILVGTNVISLENLLSRRVRSSLDKVIINMRTGKVSRKTLSPRCLDLGSINPNYA**RR**R**S**KYAYMSVMEEIPRTSGVVKIDLETGVEVASRIFGDGCYGGEPL
FV**G**KNIDNKAKENGASSDVDDEDDGYVLSYVHDEKSEESKFIV**L**DAKSPDLQIVAAVKIPRRVPYGFHGIFLSKEDLLSLKN

BoCCD4-2

MYYSSIPLPTTGHISYYNAKKKTPDLSCLYRPRERVLKLFFYEVLPSIQTYP**C**SFKFNHKMTSETTEASLPASSLSTQASKASH**F**VYS**S**F**F**NA**I**APPLEPSVDPK
NVFKGNFAPVEELPPTECLIVEGEIPTSLDGAAYIRNGTNPQYIPDRALHFFEGDGMLHSLRFSNGRAVYCSRYVKTYKFLLEKAAGAPRMPNMLSGLYGLK
DVSRFIYFYILQILIGKLNTMKGLGGANTSLAFFGKKLLALCESDLPYIIKLTEDNDIDTLERWEFDKKWMASMTAHPKVDEDTMETFAFKYCWYYPYLTFF
HFDENGVKQNEVCLLSLKQPFLIHDFAVTKRFVIFQETQLRVSLMKTMLGRGALVNYARETIPKIGVLPRYATNESDLMLFQVPGFNALHIINAWENGEDEII
VVGTNIKSIENIFSRRVNSSLDKVIINTRTGKVSMRPLSSRSLELGTINNSYAGKRNRYAYMGVMEEVLKCSGVVKIDLETGHEVASRFYGAGCFGGEPLFVK
NKHAEWADEDDGYILSYVHDENTQESKFIVLDARSPDLQVVAAVKMPRRVPYGAHGIFLSTEDISSL

BoCCD4-3

MYCYSISSPAGATIAYHE**K**TSIYT**N**ETREGMPK**I**PSFGI**L**PLIKTQQASH**L**RKLRM**T**IVKKQSTKTSSPLS**P**SFLSALASKASHFIHSSISNV**I**SPPIQPWVDPDQ
VLTGNFAPVEEMGPTECPVVEGQLPPSLNGTAYLRNGSNPQLRPRRALQYFEADGMIHSLHFSSDGRAIYSSRYVETYKYKIERKNGAATIPNFMAGFYGLI
DVARFFGLLGQILRGRLS**V**MEGFGGANTSVAFFGKKLMALCESDLPYII**T**LTEDGDIKTLKRWDFDRRVMANMTAHPKFDEDTK**E**AFAFRYNMF**C**PFLTFF
LFDENGVK**H**EDVNITSLKQPCLIHDFAITKRFVVFDETQLVFSVAKMMLGRGSIVDHNPKKIPRIGVLPRYATNDSDLKWFYVPGFNGFHLVNAWEN**E**EDEI
EILGTNVLSLGNILVKRVTTSLDKVTINMQTGEISRKVLSPRNLEFGAINSSYAGKRNRYAYFAVMEEVPKTSGVVKIDL**G**TGREVGIRFYGVG**S**FGGEPLFV
RKDAENGASPVDEDDGYVISYVHNENTEESRFLVMDAKSPELEIVAAIKMPRRVPYGFHGLFLSKEELSNIRVHTP

BoCCD4-4

MCNLICKRSVHLFDLFFSILNQFPSHSLPSKMYYSISLSNPSKIPSYHSKNSKAKSRKTILYHHKERVPRLLPSEIFPLMKTHQAPHFRKFRMVAKNETTKPSSP
PSLSFLSTLASDAAQFIYSSIFKAIAPPLDPSVDPKHVFTGNFAPVEEMVPTECPVVEGELPSSLEGAAYIRNGPNPQFLPQRALHSFEGDGMLHSLRFSNGRAI
YCSRYVKTYKYTTERDAGGPVILNFFSGFYGLIDVARYFRFIGQSMKGQVERLKGFGGANINVAFFGKKLMALCESDLPYIIDLTQDGDIETLGRWEFDMR
MFANMTAHPKVDEVTKETSAYRVNFLSPFLTFFRFDENGVKQNEVNILSMKQPSLIHDFGVTKRFMIFGETQLVLNTAKMIWGRGSLLEYRPTITPRIGILPR
YATDDSDLMWLEAPGFNPLHVLNAWENGEDEIVMVATNIKSLENILVKR**A**FTTLEKLV**T**NMRTGKLISRKSLSPKPLELGSINPSYAGKRNRYVYMAVMD
EIPRASGVVKIDVETGREVGSRFFGAGCFGGEALFVRKETENAASEDEDDGYVVTCTHDENSGD**P**MFVVMDAKSP**G**LDIVAAVQVPRRVPYGFHGLFLTR
EDLSSL

Figure S2. Predicted amino acid sequence of the isolated BoCCD1 and BoCCD4 proteins. The amino acid changes between BoCCD1 and BoCCD4 sequences and those reported in the Genebank are shown in orange. The insertion of proline in the BoCCD4-3 protein is shown in blue.
