# Supplementary material for: Identifying Bixa orellana L. New Carotenoid Cleavage Dioxygenases 1 and 4 Potentially Involved in Bixin Biosynthesis
Source: Front Plant Sci. 2022 Feb 11;13:829089. doi: 10.3389/fpls.2022.829089 (PMC8874276; doi:10.3389/fpls.2022.829089)
Supplement: Supplementary file 5 [file Data_Sheet_3.PDF]

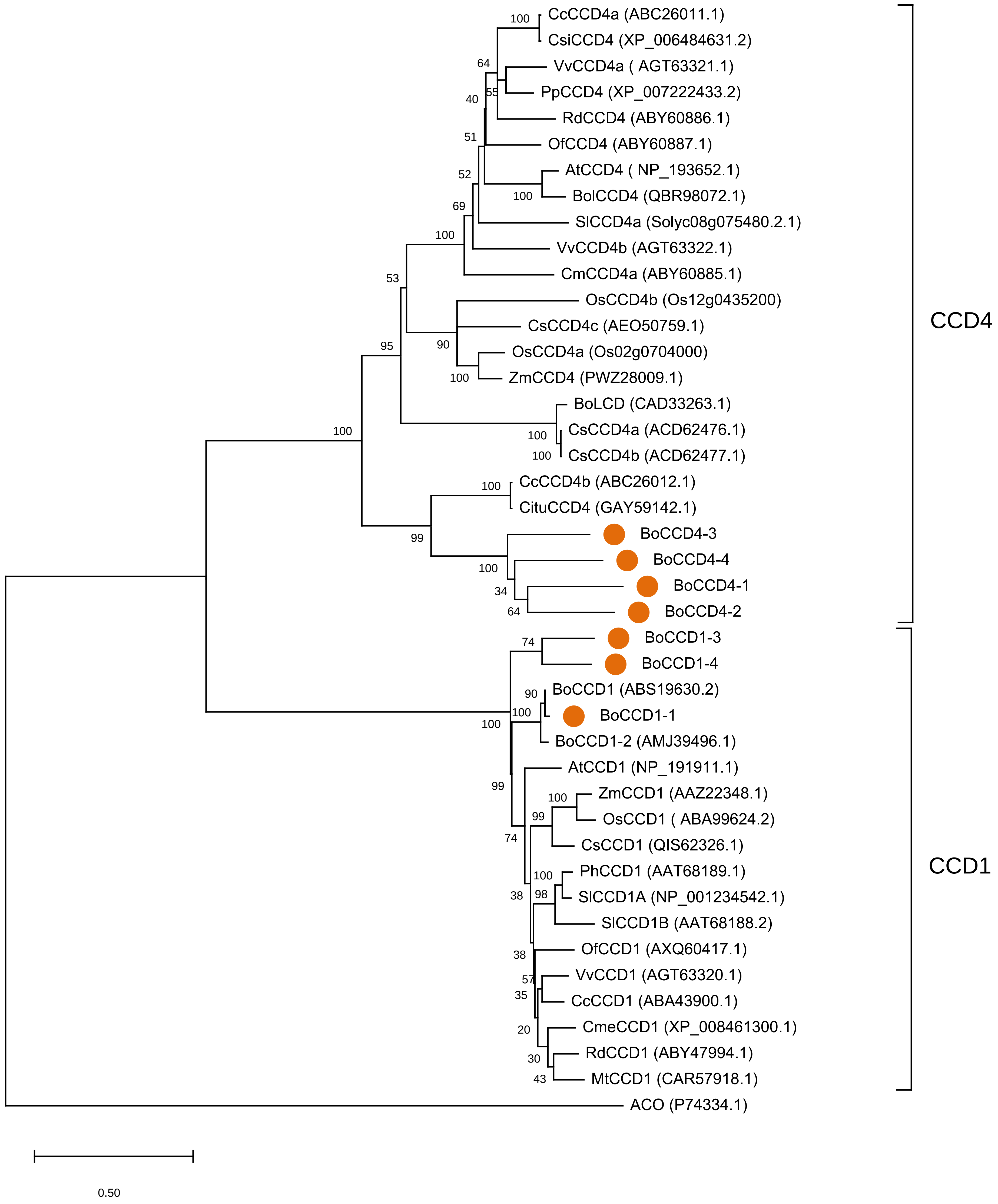

Figure S3. Phylogenetic tree of the deduced amino acid sequence of BoCCD1 and BoCCD4 proteins with other functionally characterized CCD1 and CCD4 protein sequences from different plant species. *Arabidopsis thaliana* (At), *Bixa orellana* (Bo), *Brassica oleracea* (Bol), *Citrus clementina* (Cc), *Citrus sinensis* (Csi), *Citrus unshiu* (Citu), C*hrysanthemum x morifolium* (Cm), *Coffea canephora* (Cc), *Crocus sativus* (Cs), *Cucumis melo* (Cme), *Medicago truncatula* (Mt), *Osmanthus fragrans* (Of), *Oryza sativa* (Os), *Petunia x hybrida* (Ph), *Prunus persica* (Pp), *Rosa x damascena* (Rd), *Solanum lycopersicum* (Sl), *Vitis vinifera* (Vv), *Zea mays* (Zm), and *Synechocystis sp.* (ACO).
