# Supplementary material for: Identifying Bixa orellana L. New Carotenoid Cleavage Dioxygenases 1 and 4 Potentially Involved in Bixin Biosynthesis
Source: Front Plant Sci. 2022 Feb 11;13:829089. doi: 10.3389/fpls.2022.829089 (PMC8874276; doi:10.3389/fpls.2022.829089)
Supplement: Supplementary file 6 [file Data_Sheet_4.PDF]

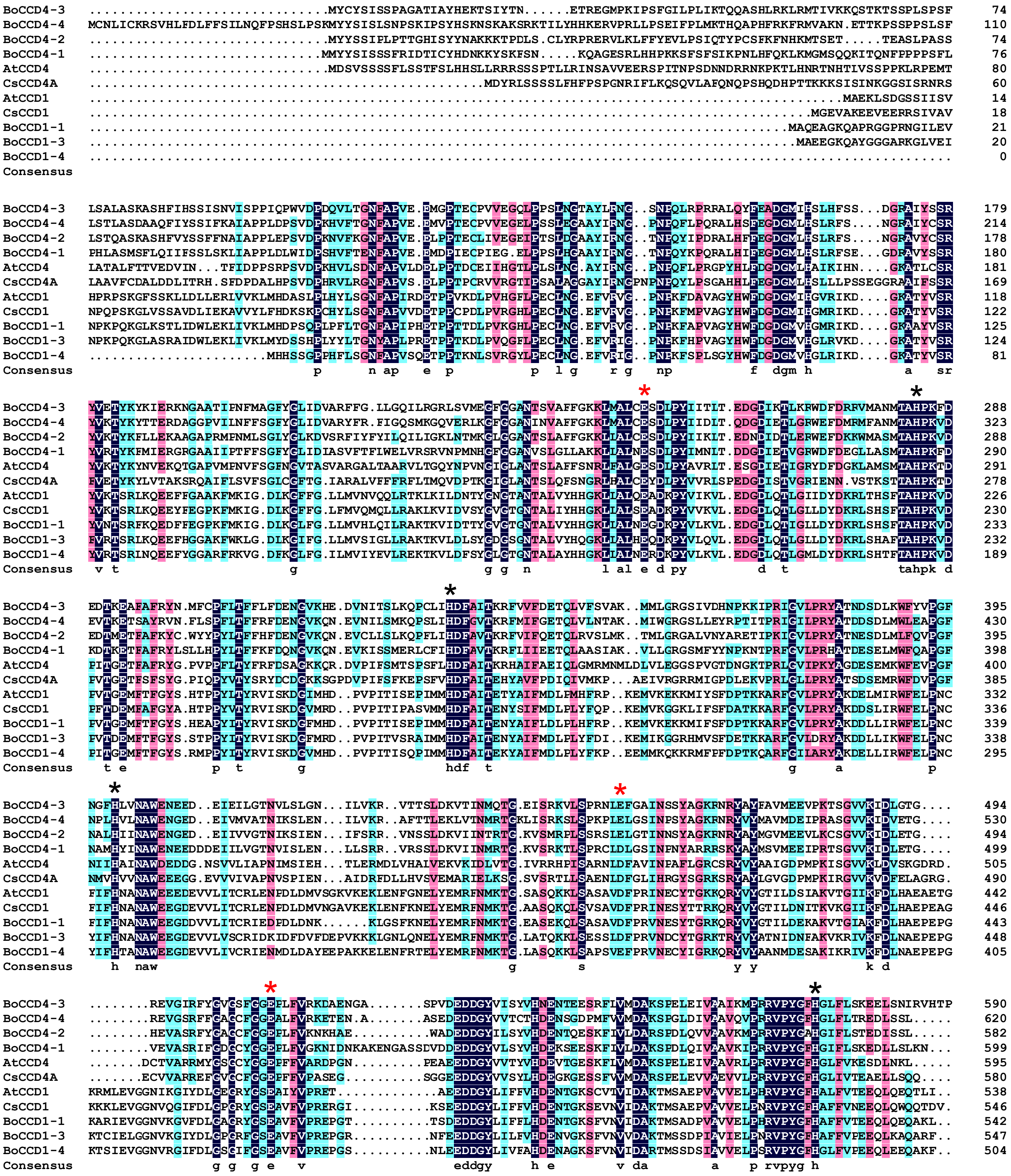

Figure S4. Alignment of the amino acid sequence of the BoCCD1 and BoCCD4 proteins and the functionally characterized BoCCD1 and BoCCD4 proteins of *A. thaliana and C. sativus.* The black asterisks indicate the four highly conserved histidine residues as an Fe2+ binding cofactor; and the red asterisks indicate the two conserved glutamic acid and one semi-conserved aspartate residues responsible for fixing the iron-linked histidine residues.
