# Supplementary material for: Identifying Bixa orellana L. New Carotenoid Cleavage Dioxygenases 1 and 4 Potentially Involved in Bixin Biosynthesis
Source: Front Plant Sci. 2022 Feb 11;13:829089. doi: 10.3389/fpls.2022.829089 (PMC8874276; doi:10.3389/fpls.2022.829089)
Supplement: Supplementary file 7 [file Data_Sheet_5.PDF]

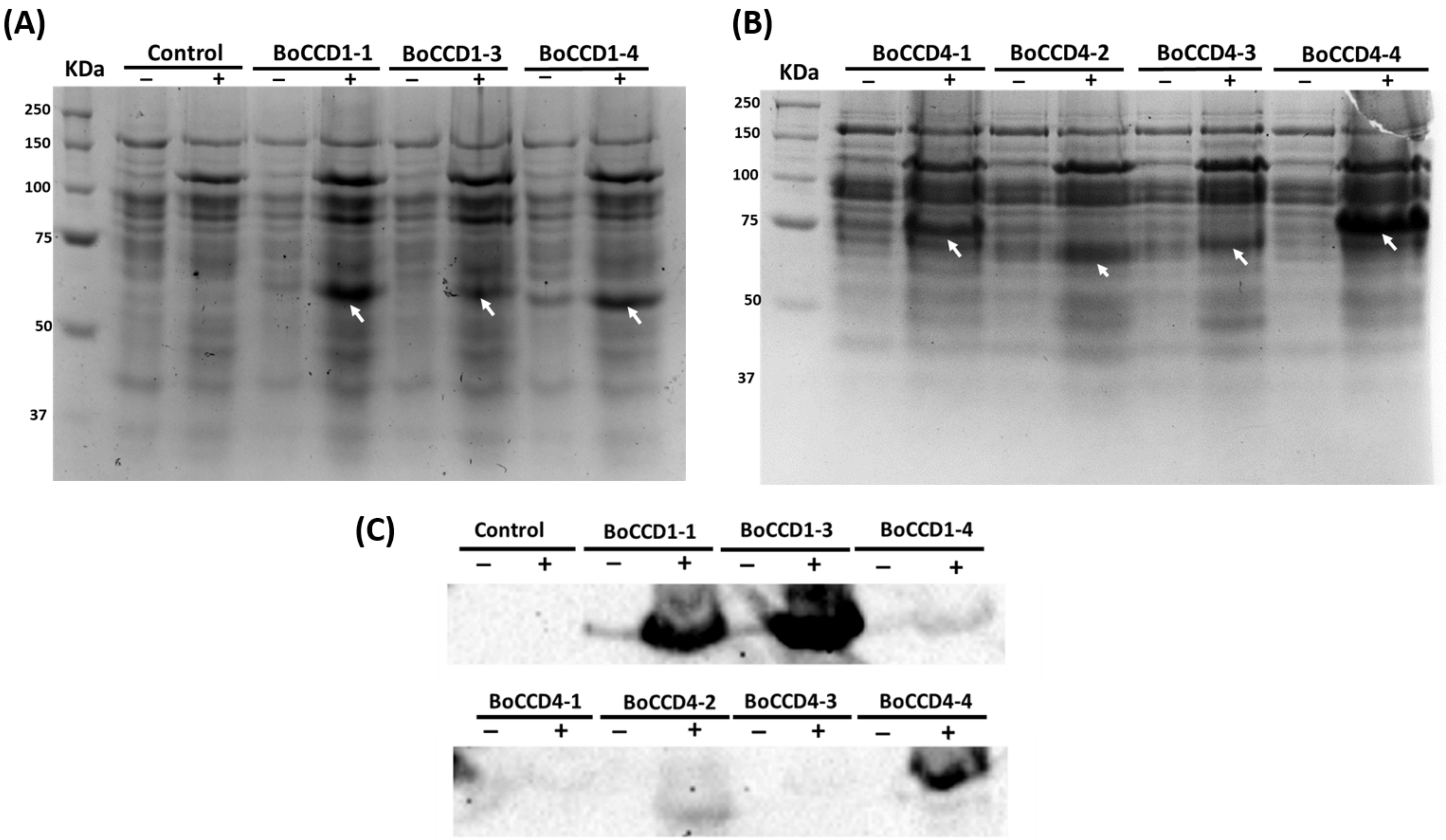

Figure S5. SDS/PAGE and blot analysis of the total protein extract of *E. coli* cells expressing BoCCD1 and BoCCD4 proteins. **A)** SDS/PAGE of total protein extract of *E. coli* cells expressing control, BoCCD1-1, BoCCD1-3, and BoCCD1-4 proteins. **B)** SDS/PAGE of the total protein extract of *E. coli* cells expressing BoCCD4-1, BoCCD4-2, BoCCD4-3, and BoCCD4-4. **C)** Blot analysis of BoCCD1-1, BoCCD1-3, BoCCD1-4, BoCCD4-1, BoCCD4-2, BoCCD4-3, and BoCCD4-4 proteins using anti-His antibody. The extracts were obtained from uninduced cells (−) or after being induced (+) for 20 h. The amount of protein loaded was determined by OD_{600}. The empty pDEST17 vector was used as a negative control. Precision Plus protein was used as a molecular mass marker.
