# Supplementary material for: Identifying Bixa orellana L. New Carotenoid Cleavage Dioxygenases 1 and 4 Potentially Involved in Bixin Biosynthesis
Source: Front Plant Sci. 2022 Feb 11;13:829089. doi: 10.3389/fpls.2022.829089 (PMC8874276; doi:10.3389/fpls.2022.829089)
Supplement: Supplementary file 8 [file Data_Sheet_6.PDF]

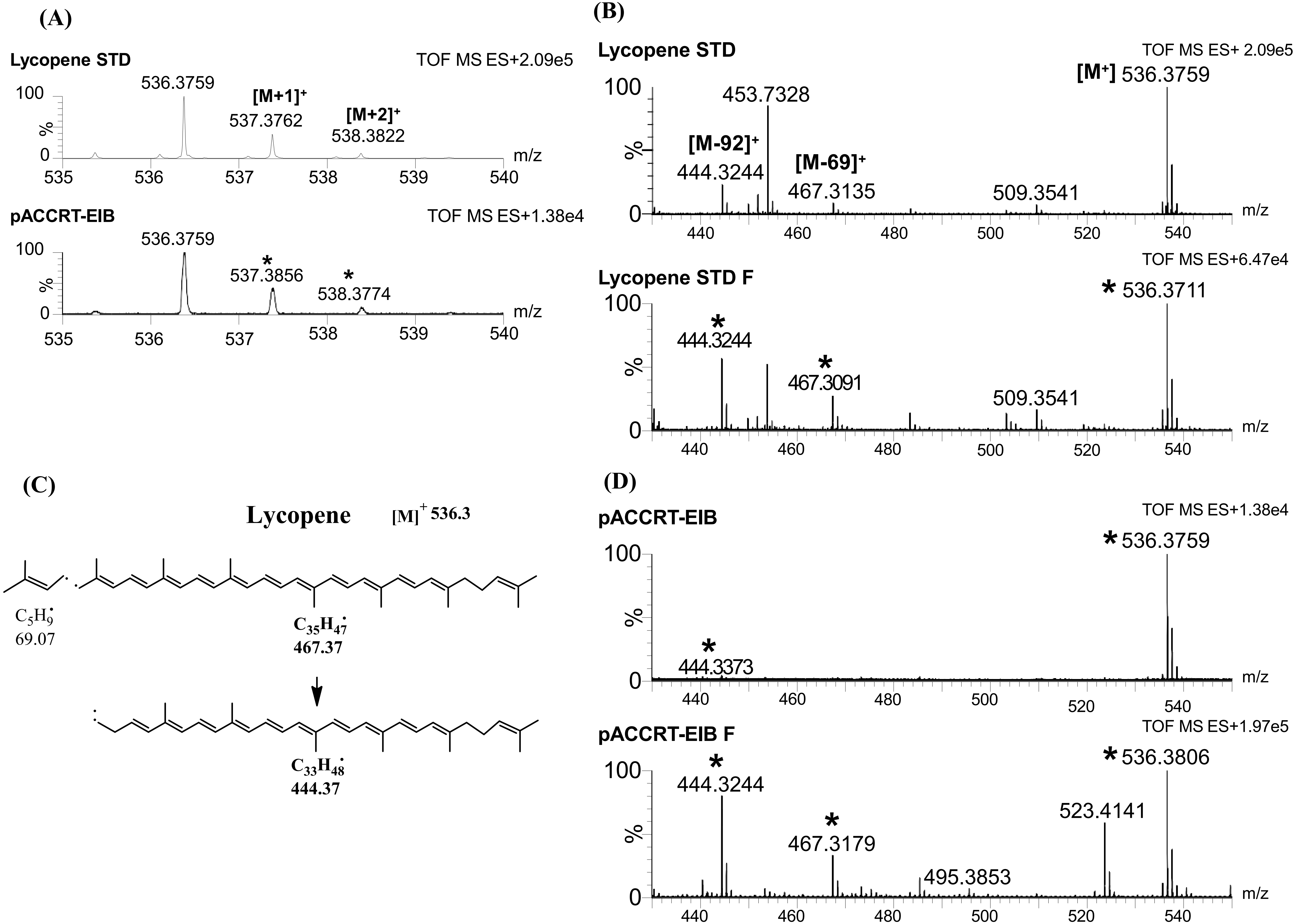

Figure S6. MS and MS/MS spectra of the lycopene standard, and lycopene produced by pCCART-IEB *E. coli* cells. **A)** MS spectra of lycopene standard, and lycopene produced by pCCART-IEB *E. coli* cells. **B)** MS and MS/MS spectra of lycopene standard (*m/z* 536.3), and **D)** lycopene produced by pCCART-IEB *E. coli* cells. **C)** Predicted ion fragmentation of lycopene.
