# Supplementary material for: Identifying Bixa orellana L. New Carotenoid Cleavage Dioxygenases 1 and 4 Potentially Involved in Bixin Biosynthesis
Source: Front Plant Sci. 2022 Feb 11;13:829089. doi: 10.3389/fpls.2022.829089 (PMC8874276; doi:10.3389/fpls.2022.829089)
Supplement: Supplementary file 9 [file Data_Sheet_7.PDF]

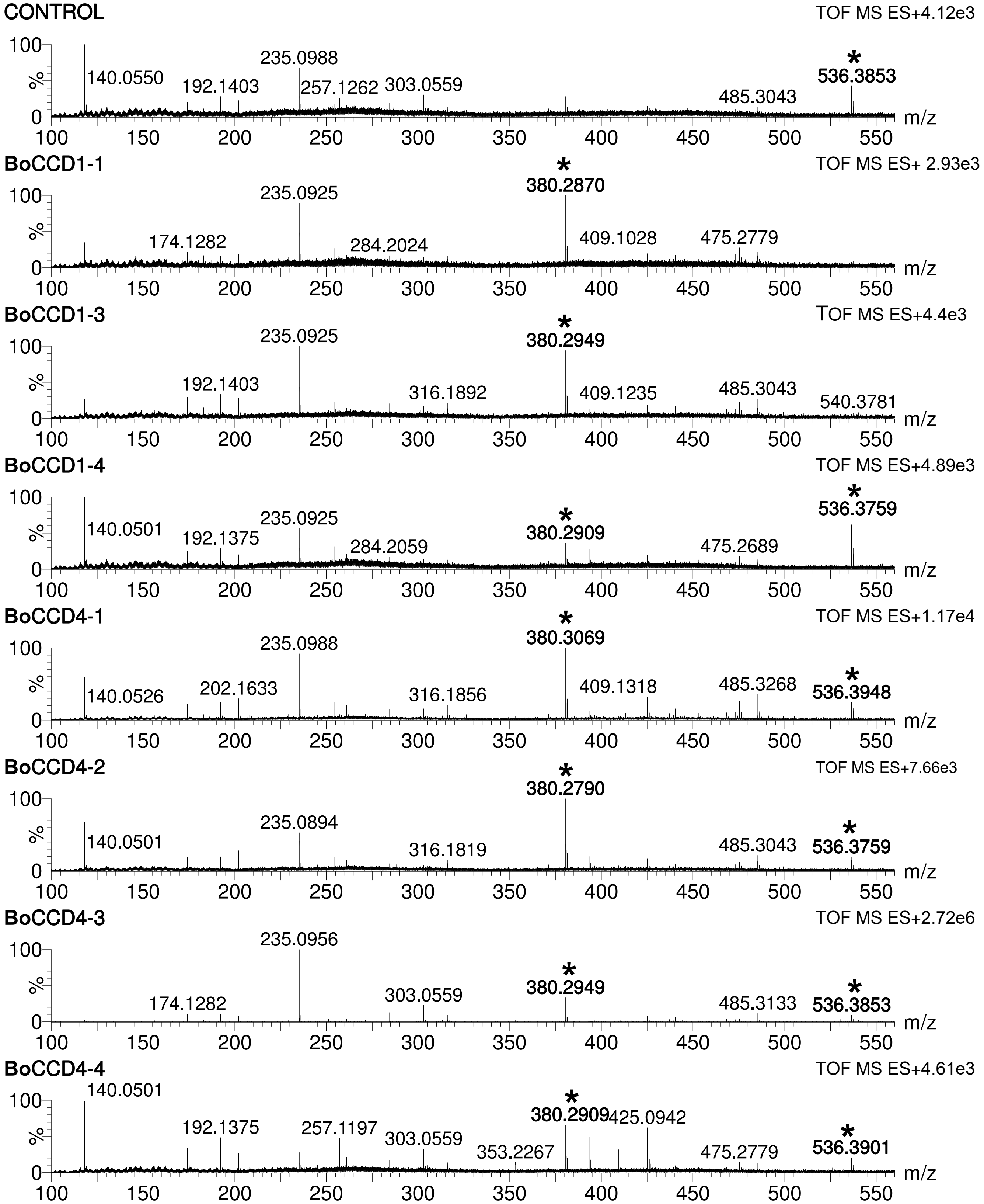


**Figure S7.** ESI-MS spectra of the bacterial extracts obtained from the expression of BoCCD1 and BoCCD4 proteins in pCCART-IEB *E. coli* cells. The empty vector was used as a control.
