# Supplementary material for: Identifying Bixa orellana L. New Carotenoid Cleavage Dioxygenases 1 and 4 Potentially Involved in Bixin Biosynthesis
Source: Front Plant Sci. 2022 Feb 11;13:829089. doi: 10.3389/fpls.2022.829089 (PMC8874276; doi:10.3389/fpls.2022.829089)
Supplement: Supplementary file 10 [file Data_Sheet_8.PDF]

(A)

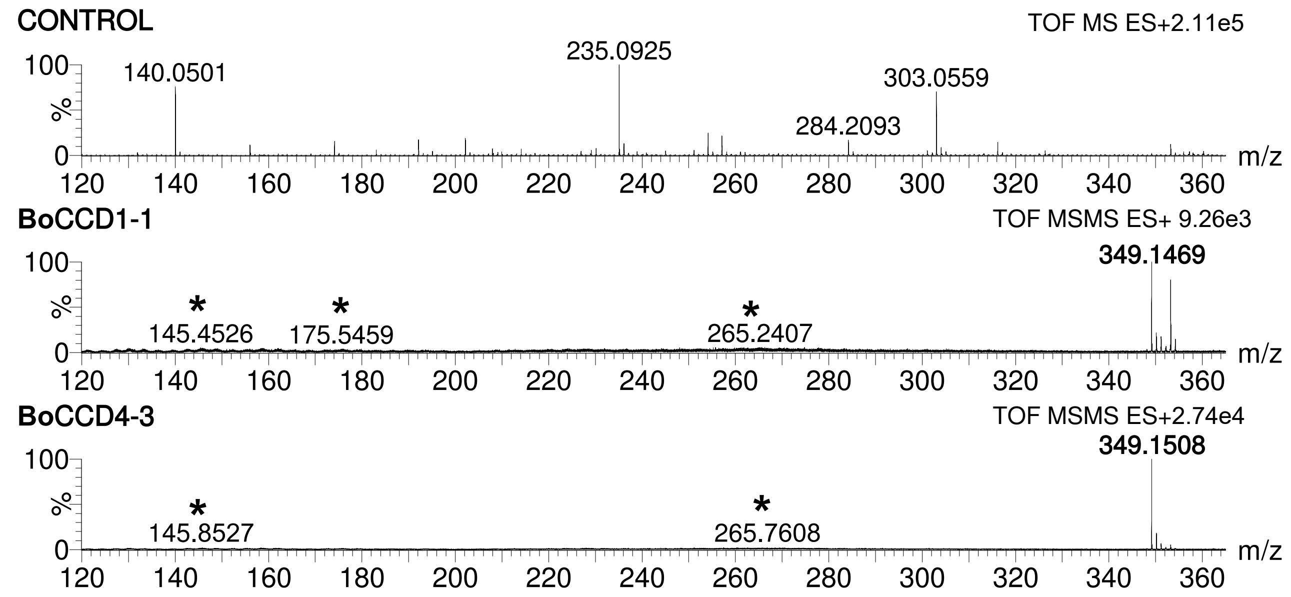

(B)

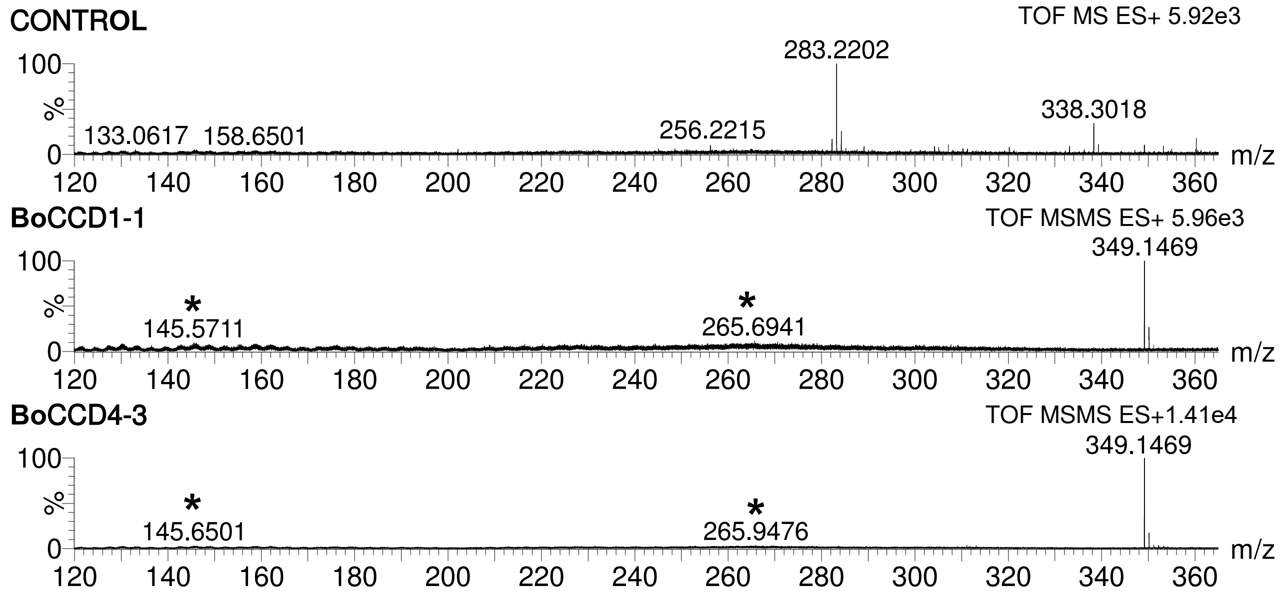

Figure S8. MS/MS spectra of bixin aldehyde (*m/z* 349.1) of the extracts obtained from the **A)** *in vivo*, and **B)** i*n vitro* expression of the BoCCD1-1 and BoCCD4-3 proteins.
