# Supplementary material for: Identifying Bixa orellana L. New Carotenoid Cleavage Dioxygenases 1 and 4 Potentially Involved in Bixin Biosynthesis
Source: Front Plant Sci. 2022 Feb 11;13:829089. doi: 10.3389/fpls.2022.829089 (PMC8874276; doi:10.3389/fpls.2022.829089)
Supplement: Supplementary file 11 [file Data_Sheet_9.PDF]

Bixin aldehyde

$[M]^+$ 348

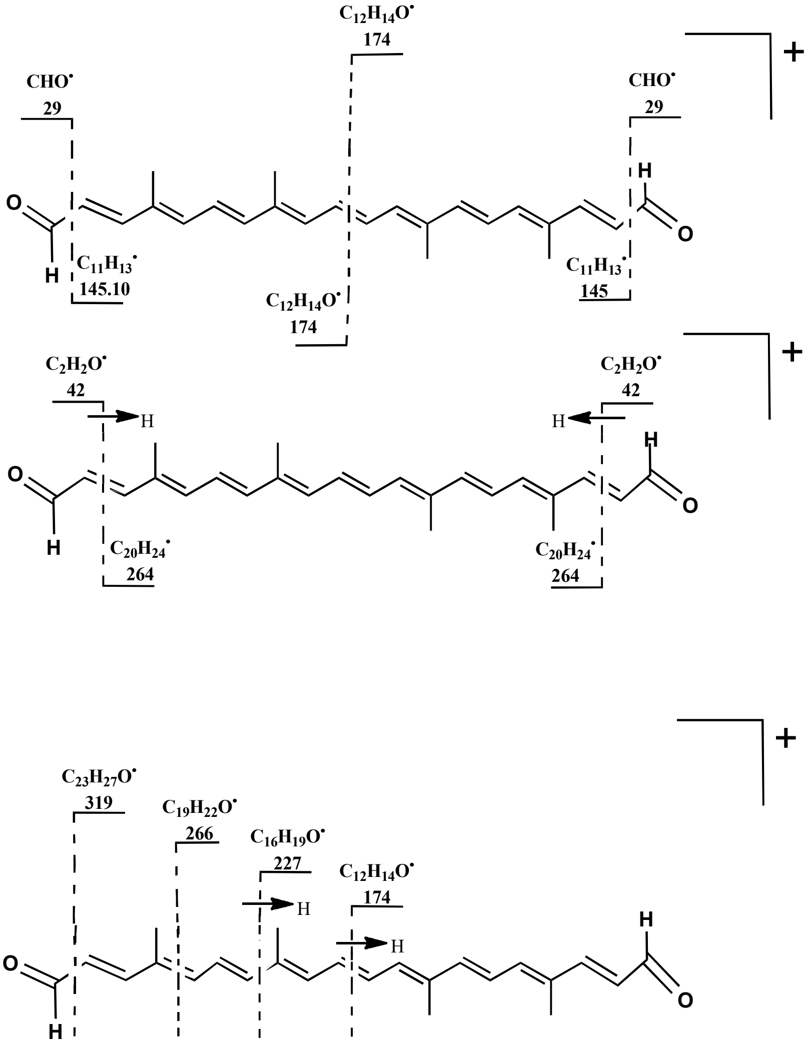

Figure S9. Predicted ion fragments generated in the ESI-MS/MS fragmentation of bixin aldehyde.
