# Supplementary material for: Identifying Bixa orellana L. New Carotenoid Cleavage Dioxygenases 1 and 4 Potentially Involved in Bixin Biosynthesis
Source: Front Plant Sci. 2022 Feb 11;13:829089. doi: 10.3389/fpls.2022.829089 (PMC8874276; doi:10.3389/fpls.2022.829089)
Supplement: Supplementary file 14 [file Data_Sheet_12.PDF]

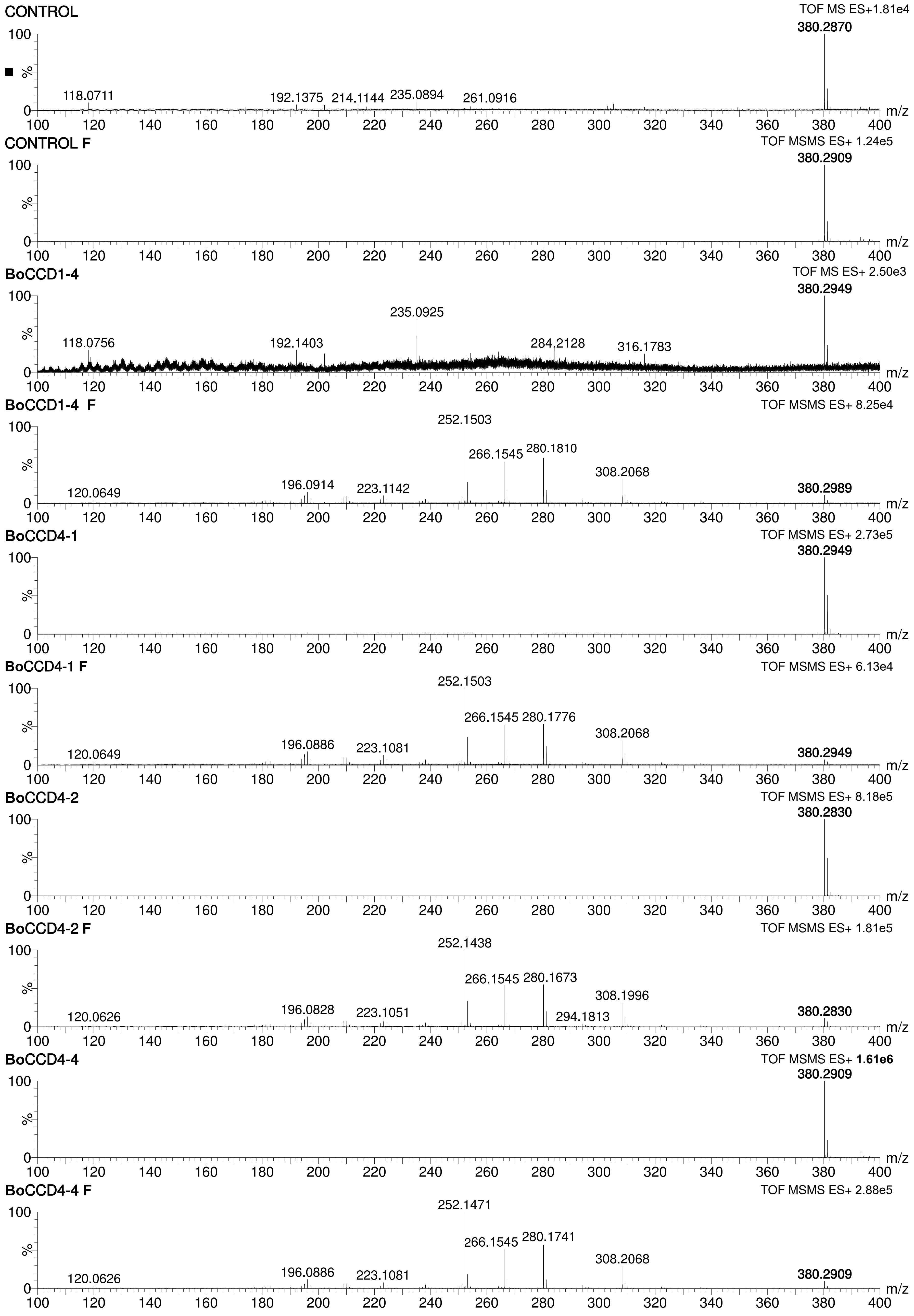

Figure S12. MS and MS/MS spectra of norbixin (*m/z* 380.2) of the extracts from the expression of the BoCCD1-4, BoCCD4-1, BoCCD4-2 and BoCCD4-4 proteins in pCCART-IEB *E. coli* cells. The empty vector was used as a control.
