# Supplementary material for: Identifying Bixa orellana L. New Carotenoid Cleavage Dioxygenases 1 and 4 Potentially Involved in Bixin Biosynthesis
Source: Front Plant Sci. 2022 Feb 11;13:829089. doi: 10.3389/fpls.2022.829089 (PMC8874276; doi:10.3389/fpls.2022.829089)
Supplement: Supplementary file 15 [file Data_Sheet_13.PDF]

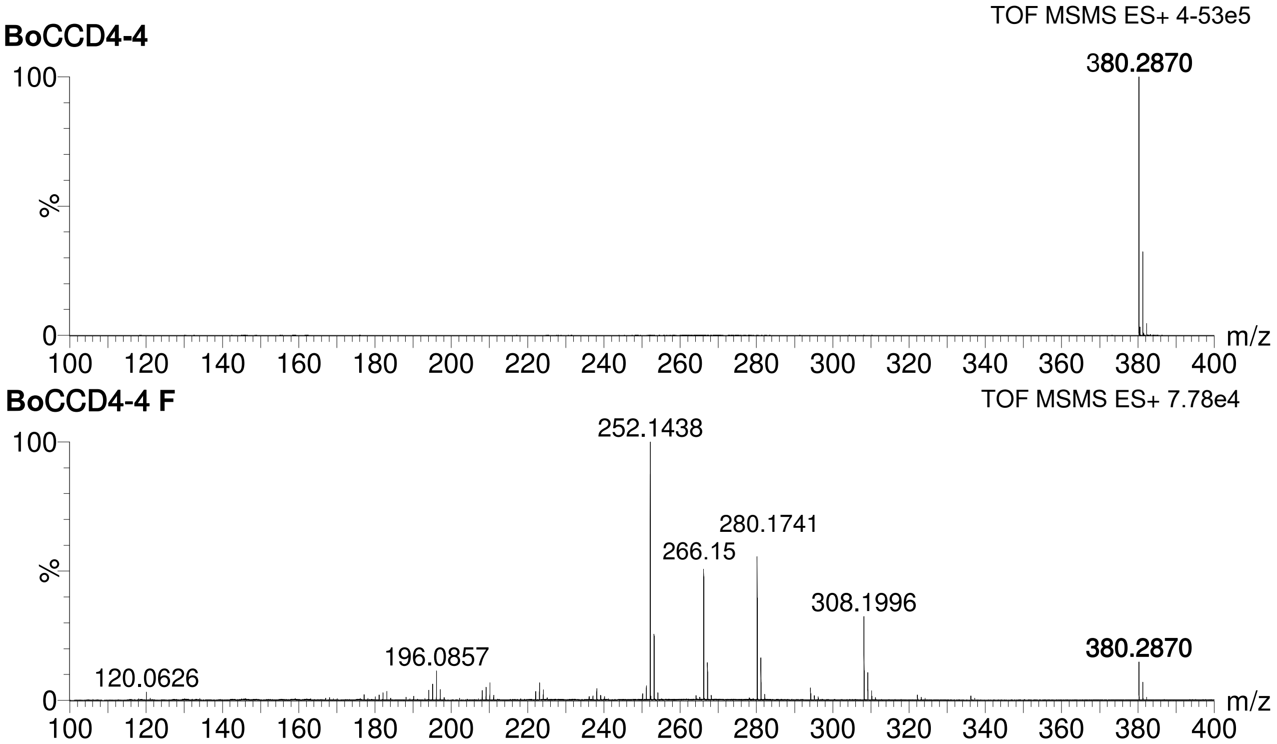

Figure S14. MS, and MS/MS spectra of norbixin (*m/z 3*80.2) of the extract from the i*n vitro* enzymatic reaction of the BoCCD4-4 protein.
